# Fetuin-A is an immunomodulator and a potential therapeutic option in BMP4-dependent heterotopic ossification and associated bone mass loss

**DOI:** 10.1038/s41413-022-00232-x

**Published:** 2022-10-27

**Authors:** Chen Kan, Jiazhao Yang, Haitao Fan, Yuanjuan Dai, Xingxing Wang, Rui Chen, Jia Liu, Xiangyue Meng, Wei Wang, Guiling Li, Jiao Zhou, Ya Zhang, Wanbo Zhu, Shiyuan Fang, Haiming Wei, Hong Zheng, Siying Wang, Fang Ni

**Affiliations:** 1 Department of Pathophysiology, School of Basic Medical Sciences, Anhui Medical University, Hefei, China; 2 Department of Hematology, The First Affiliated Hospital of USTC, The CAS Key Laboratory of Innate Immunity and Chronic Disease, School of Basic Medical Sciences, Division of Life Sciences and Medicine, University of Science and Technology of China, Hefei, China; 3 Institute of Immunology, University of Science and Technology of China, Hefei, China; 4 Department of Orthopaedics, The First Affiliated Hospital of USTC, Hefei, China; 5 Department of Orthopaedics, Fuyang Hospital of Anhui Medical University, Fuyang, China

**Keywords:** Bone, Metabolic diseases

## Abstract

Heterotopic ossification (HO) is the abnormal formation of bone in extraskeletal sites. However, the mechanisms linking HO pathogenesis with bone mass dysfunction remain unclear. Here, we showed that mice harboring injury-induced and BMP4-dependent HO exhibit bone mass loss similar to that presented by patients with HO. Moreover, we found that injury-induced hyperinflammatory responses at the injury site triggered HO initiation but did not result in bone mass loss at 1 day post-injury (dpi). In contrast, a suppressive immune response promoted HO propagation and bone mass loss by 7 dpi. Correcting immune dysregulation by PD1/PDL1 blockade dramatically alleviated HO propagation and bone mass loss. We further demonstrated that fetuin-A (FetA), which has been frequently detected in HO lesions but rarely observed in HO-adjacent normal bone, acts as an immunomodulator to promote PD1 expression and M2 macrophage polarization, leading to immunosuppression. Intervention with recombinant FetA inhibited hyperinflammation and prevented HO and associated bone mass loss. Collectively, our findings provide new insights into the osteoimmunological interactions that occur during HO formation and suggest that FetA is an immunosuppressor and a potential therapeutic option for the treatment of HO.

## Introduction

Heterotopic ossification (HO) is a potentially severe pathologic process defined as the pathological growth of extraskeletal bone^1–3^. HO can be acquired or hereditary. The most common type is acquired HO (aHO), which can be caused by injury or surgery. Patients with aHO often develop chronic pain, unhealed wounds, and restricted joint motion, leading to a diminished quality of life. Although hospitalized patients with HO are common, the underlying mechanisms that lead to HO formation and the associated complications are still largely unknown.

As in normal skeletal morphogenesis, HO can form through both intramembranous and endochondral ossification processes. Two major types of cells contribute to bone homeostasis^4^. Osteoblasts, as bone-forming cells, secrete abundant proteins and form osteoids, which mineralize to become bone^5^. Osteoclasts resorb or break down bone by secreting acids and collagenases^6^. The equilibrium between osteoblasts and osteoclasts maintains bone tissue. However, if this delicate balance is disrupted, bone diseases, such as bone fracture, arthritis, and osteoporosis, will occur^7,8^. To date, the bone structure during injury-induced HO formation has remained unexplored. In addition, the link between HO pathogenesis and the effect on bone mass has not been established. Furthermore, the potential mechanisms linking HO pathogenesis with bone mass dysfunction remain unclear.

Recent studies have shed light on the roles of the immune system in HO initiation and propagation^9,10^. Several immune cell types in both the innate immune system and adaptive immune system, such as macrophages, neutrophils, mast cells, and lymphocytes, have been shown to have potential roles in ectopic bone formation through various mechanisms^11,12^. Furthermore, dysregulated immune responses are closely linked with HO and associated bone diseases^13^. Although many lines of evidence suggest an immunological contribution to HO disorders, little information about the dynamic changes in the immune response in HO lesions has been reported. In addition, definite targets allowing the design of treatments to inhibit or prevent HO remain to be identified.

In the present study, we show that injury-induced HO is accompanied by bone mass loss in mice and humans. The injury-induced early inflammatory response (1 day after injury) at the injury site was shown to trigger HO but not bone mass loss, whereas the later (7 days after injury) suppressive immune response promoted HO, leading to local bone mass loss. Furthermore, we identified that Fetuin-A (FetA), which is detected frequently in HO lesions but rarely in HO-adjacent normal bone, stimulates PD1 to suppress the inflammatory response and that intervention with FetA inhibited HO and bone mass loss. These data reveal a mechanism underlying injury-induced HO pathogenesis and associated bone loss and suggest that FetA may be a potential option to prevent HO.

## Results

### Injury-induced HO is accompanied by bone loss in mice and humans

**Fig. 1:**
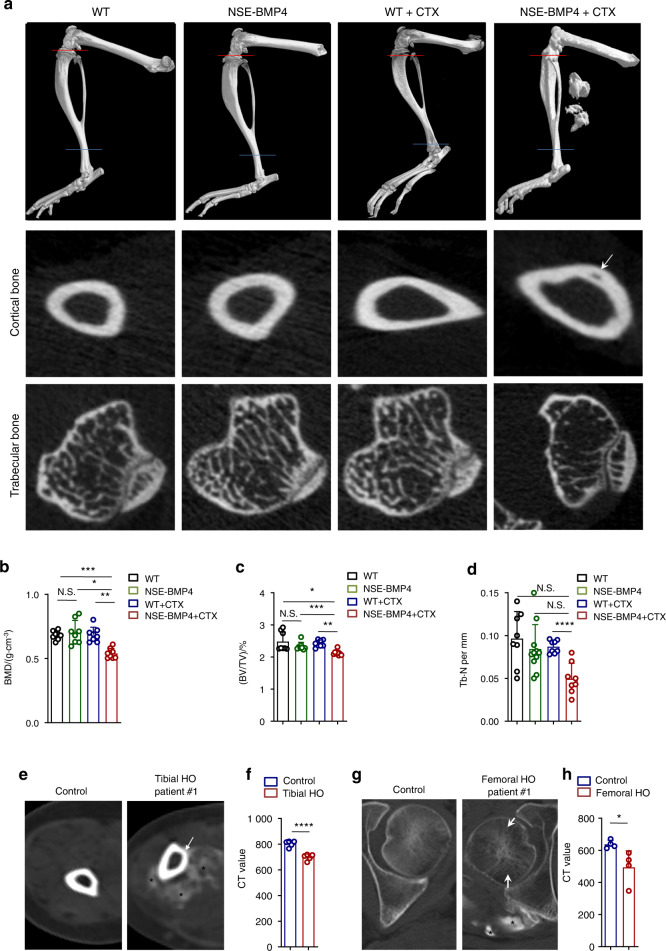
Mice harboring injury-induced HO exhibit bone loss similar to that in patients with HO. **a** Representative microCT images of the hindlimbs of the uninjured WT and NSE-BMP4 mice as well as the injured WT and NSE-BMP4 mice at 14 dpi. Red and blue lines indicate the transverse regions of selected cortical and trabecular bone in the tibia, respectively. Arrows indicate bone loss.Statistical analysis of BMD (**b**), BV/TV (**c**) and Tb. N (**d**) of the WT and NSE-BMP4 mice with and without tibial muscle injury; *n* = 8 per group. Data are presented as the mean ± s.d. of biological replicates. ^*^P < 0.05, ^**^P < 0.01, ^***^P < 0.001, ^****^P < 0.000 1. N.S. indicates no significance (unpaired two-tailed *t* test). **e** Representative CT image of a transverse tibia bone from a patient with tibial HO and a healthy control. Arrows indicate bone mass loss. Asterisks indicate HO. **f** Statistical analysis of CT values from the entire tibia adjacent to the HO and the healthy controls. *n* = 4–6 per group. Data are presented as the mean ± s.d. of biological replicates. ^****^P < 0.000 1 (unpaired two-tailed *t* test). **g** Representative CT image of a transverse tibia bone from a patient with femoral HO and a healthy control. Arrows indicate bone loss. Asterisks indicate HO. **h** Statistical analysis of CT values from the entire femur adjacent to the HO and the healthy controls. *n* = 5–6 per group. Data are presented as the mean ± s.d. of biological replicates. ^*^P < 0.05 (unpaired two-tailed *t* test)

HO is characterized by the presence of mature osteoids and marrow in soft tissues, leading to restricted mobility^14^. We investigated genetically modified mice (NSE-BMP4) that had been previously shown to efficiently recapitulate HO upon injury^15^. We confirmed the successful HO model induction 2 weeks after injury to the tibial muscle, and microCT scans showed that the NSE-BMP4 mice developed tibial HO, whereas no extra ossification was observed in the injured WT mice (Fig. 1a). Without injury model induction, neither the WT mice nor the NSE-BMP4 mice developed HO (Fig. 1a). We next examined the tibial bone mass in the WT and NSE-BMP4 mice with and without injury. MicroCT images showed that cortical and trabecular bone mass loss was present in the HO mouse model group but not in the other groups (Fig. 1a). Hematoxylin and eosin (HE) staining and safranin O staining showed that only the HO model mice displayed cortical bone mass loss (Fig. S1a, b). Safranin O staining also revealed ectopic endochondral bone formation in the injured muscle of the NSE-BMP4 mice, confirming the availability of this model to investigate HO pathogenesis (Fig. S1b). We further used CT analysis to quantify the entire tibial bone structure in detail. In the absence of injury, no significant difference in bone mineral density (BMD) was observed between the NSE-BMP4 and WT mice (Fig. 1b). However, following the development of HO, tibial BMD values were significantly decreased in the HO model mice compared to the injured WT mice (Fig. 1b). Consistently, the ratio of bone volume to tissue volume (BV/TV) and the number of trabeculae (Tb. N) were markedly reduced in the HO model mice (Fig. 1c, d). No differences were detected in the ratio of bone surface area to bone volume (BS/BV), trabecular separation (Tb. Sp), or in trabecular thickness (Tb. Th) (Fig. S1b).

Since HO begins some distance from normal bone and eventually fuses to it^16^, we next examined the anatomical connection between HO and bone. We again injured the tibial muscle of the NSE-BMP4 mice and measured the BMD of HO-adjacent tibia at different time points post-injury (e.g., at 0, 1, 3, 7 and 14 dpi). We found that bone mass loss occurred at 7 dpi, while HO did not fuse with bone (Fig. S2a, b). HE staining also showed HO-adjacent bone erosion initiated at 7 dpi (Fig. S3). Consistently, cathepsin K (CTSK) staining (Fig. S4a, b) and tartrate resistant acid phosphatase (TRAP) staining (Fig. S5a, b) showed that osteoclasts in the HO-adjacent tibia (including cortical and trabecular bone) were initially increased at 7 dpi, suggesting that bone mass loss occurred earlier than HO-bone fusion. Moreover, we found that the extent of bone mass loss was more severe at 14 dpi than at 7 dpi (Fig. S2a, b and Fig. S3). The populations of CTSK^+^ and TRAP^+^ cells were further increased at 14 dpi compared to those at 7 dpi (Fig. S4a, b and Fig. S5a, b). We observed that some HO had fused to bone (Fig. S6a and Supplementary Movies 1–3). We then compared the tibial BMD among the non-HO group, nonfusion HO group and fusion HO group. The BMD of the tibia adjacent to HO in the fusion-HO group was significantly lower than that in the nonfusion HO group, suggesting that HO-bone fusion enhanced HO-induced bone mass loss (Fig. S6a, b). We next investigated the effects of tibial HO on the femur. We specifically induced muscle injury adjacent to the tibia, femur, or both sites. Strikingly, tibial HO affected only the tibial BMD, while the femoral BMD was affected by only femoral HO (Fig. S7a, b).

Extending these initial findings suggesting an apparent connection between HO and bone mass loss in the mouse model, we recruited 9 patients with aHO (4 femoral cases and 5 tibial cases) and healthy control individuals and used CT to assess their bones. As in the mouse model, we detected significant bone mass loss in HO-adjacent bones (Fig. 1e–h and Fig. S7c). Furthermore, no obvious tibial abnormalities were detected in the patients with femoral HO, while the patients with tibial HO had no femur abnormalities (Fig. S7d). Taken together, these results reveal that HO is accompanied by bone mass loss and that HO-bone fusion enhances bone mass loss.

### The injury-induced hyperinflammatory response drives BMP4-dependent HO pathogenesis but not bone mass loss

**Fig. 2:**
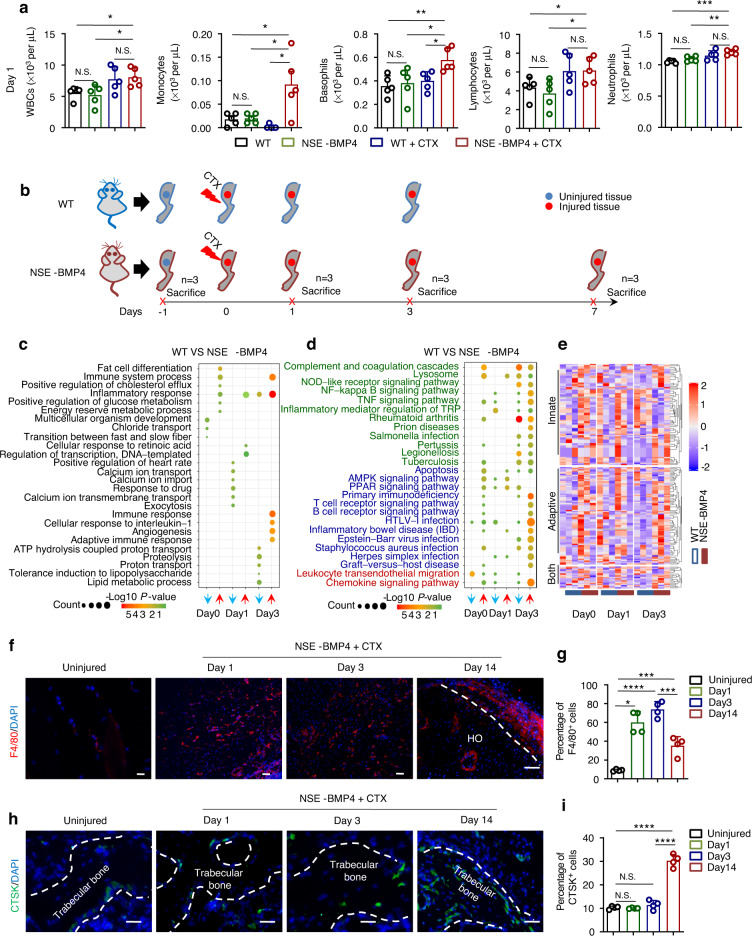
The injury-induced inflammatory response at the site of injury triggers HO but not bone loss at 1 dpi. **a** White blood cells (WBCs), monocytes, basophils, lymphocytes, and neutrophils in the peripheral blood of six- to eight-week-old WT and NSE-BMP4 mice at 1 day after injury. (*n* = 4-5 per group). Data are presented as the mean ± s.d. of biological replicates. ^*^P < 0.05, ^**^P < 0.01, ^***^P < 0.001 (unpaired two-tailed *t* test). **b** Schematic outline of *t*he study design depicting the workflow of target tissue collection at different time points for RNA-seq. **c** GO enrichment analysis of differentially expressed genes (DEGs) in the tibial muscle of the NSE-BMP4 mice (*n* = 3 per group). Enrichment analysis of immune cell genes in the NSE-BMP4 mice, presented in a KEGG pathway dot plot (**d**) and heatmap (**e**) (*n* = 3 per group). IF staining images (**f**) and statistical analysis (**g**) of F4/80^+^ macrophages in HO lesions after CTX injury. The white dotted line outlines the region of mature HO. Scale bar, 200 μm (*n* = 3 per group, *n* = 3 ROI/mouse). Data are presented as the mean ± s.d. of biological replicates. ^*^P < 0.05, ^***^P < 0.001, ^****^P < 0.000 1. N.S. indicates no significance (unpaired two-tailed *t* test). Immunostaining images (**h**) and statistical analysis (**i**) of CTSK^**+**^ osteoclasts in bone from the NSE-BMP4 mice after CTX injury. The white dotted line outlines the region of CTSK^+^ cells. Scale bar, 200 μm (*n* = 3 per group, *n* = 3 ROI/mouse). Data are presented as the mean ± s.d. of biological replicates. ^****^P < 0.000 1. N.S. indicates no significance (unpaired two-tailed *t* test)

Inflammation is a precondition for HO and is a risk factor for bone loss^17,18^. Accordingly, we hypothesized that the inflammatory response(s) following tibial muscle injury may contribute to the initiation of HO and bone loss. Following muscle injuries in mice, inflammation typically develops rapidly but often resolves within approximately one week^19^. We thus repeated the injury procedure targeting the tibial muscle of the WT and NSE-BMP4 mice and evaluated the number of circulating white blood cells (WBCs) as an indicator of systemic immune reactions. No significant differences were found in the total WBCs between the uninjured WT and NSE-BMP4 mice (Fig. 2a). However, 1-day post-injury, we observed that the absolute numbers of monocytes and basophils in NSE-BMP4 mouse blood were increased significantly compared to those in WT mouse blood (Fig. 2a). At Day 3 post-injury, there were still apparently increased counts of total WBCs in the NSE-BMP4 mice, but the differences compared to the injured WT mice were not statistically significant (Fig. S8a). To determine any clinical relevance of the increased blood cell counts, we retrospectively analyzed the peripheral WBCs of trauma patients from 2017 to 2019. We collected clinical data for a total of 221 patients with muscle injury or hip fracture and found that 10 patients developed HO. We then screened the WBC data meeting the requirement that patients did not receive anti-inflammatory drugs or other interventions at the time of admission (i.e., on the day of trauma). Eleven non-HO patients with traumatic injury (Table S1) and 10 patients with aHO (Table S2) were then analyzed. Both the total WBC count and the numbers of basophils, neutrophils and monocytes were increased in the patients with aHO compared to the patients with muscle injury or fracture who did not develop HO (Fig. S8b).

We next investigated the immune response in local muscle tissue of the WT and NSE-BMP4 mice with or without injury. There were no significant differences in the expression of inflammatory cytokines in the muscle between the uninjured WT and NSE-BMP4 mice (Fig. S8c). We further performed an RNA-sequencing analysis to examine the immune response at the injury site over 3 days post-injury in the WT and NSE-BMP4 mice (Fig. 2b). The expression of *Bmp4* and *Smad9*, which are the key elements of the BMP signaling pathway, was significantly higher in the injured tibial muscle of the NSE-BMP4 mice than in the WT mice, supporting the utility of our HO model mice (Fig. S9a–i). Principal component analysis (PCA) of the transcriptomic data readily separated temporal trends in the postinjury expression profiles of the NSE-BMP4 and WT mice (Fig. S10a). Consistent with a previous study^19^, the RNA-sequencing assay revealed enrichment of genes annotated to “immune system process and inflammatory response” on postinjury Day 1, but this enrichment was attenuated from Day 3 onward (Fig. S10b, d). Moreover, GO analysis revealed enrichment of genes with “inflammatory response”-related functional annotations in NSE-BMP4 mice on postinjury Days 1 and 3 (Fig. 2c). KEGG pathway analysis indicated that the injured NSE-BMP4 mice had apparent downregulation of genes involved in some innate immune pathways but had upregulation of genes involved in some adaptive immune pathways (Fig. 2d, e). Together, the increased WBC counts and enrichment of genes annotated to “inflammatory response” at the injury site in the NSE-BMP4 mice suggested that a hyperinflammatory response could be a driver of HO pathogenesis.

Previous work has shown that monocytes can differentiate into macrophages and osteoclasts^20^, cell types that function in promoting HO^12^ and bone loss, respectively^8^. We thus speculated that increased levels of hyperinflammation-responsive monocytes in the blood of the NSE-BMP4 mice could contribute to lesional macrophage infiltration and bone osteoclast activation. We repeated tibial muscle injury in the NSE-BMP4 mice, and microCT and immunostaining analysis revealed that the BMD and osteoclasts of the tibia of the NSE-BMP4 mice did not change within 3 days of injury (Fig. S2a, b, Fig. S4a, b and Fig. 5a, b). Immunofluorescence (IF) staining of injury sites revealed the accumulation of F4/80^+^ macrophages at HO lesions in the injured NSE-BMP4 mice (Fig. 2f, g); no obvious changes were detected in CTSK^+^ osteoclasts in the trabecular bone of the tibia adjacent to HO lesions (Fig. 2h, i). Collectively, these results indicate that injury-induced hyperinflammation is closely associated with HO initiation but not with the proliferation of osteoclasts in HO-adjacent bone.

### Compounds with anti-inflammatory activity, including rapamycin and ebselen, prevent BMP4-dependent HO and subsequent bone loss

**Fig. 3:**
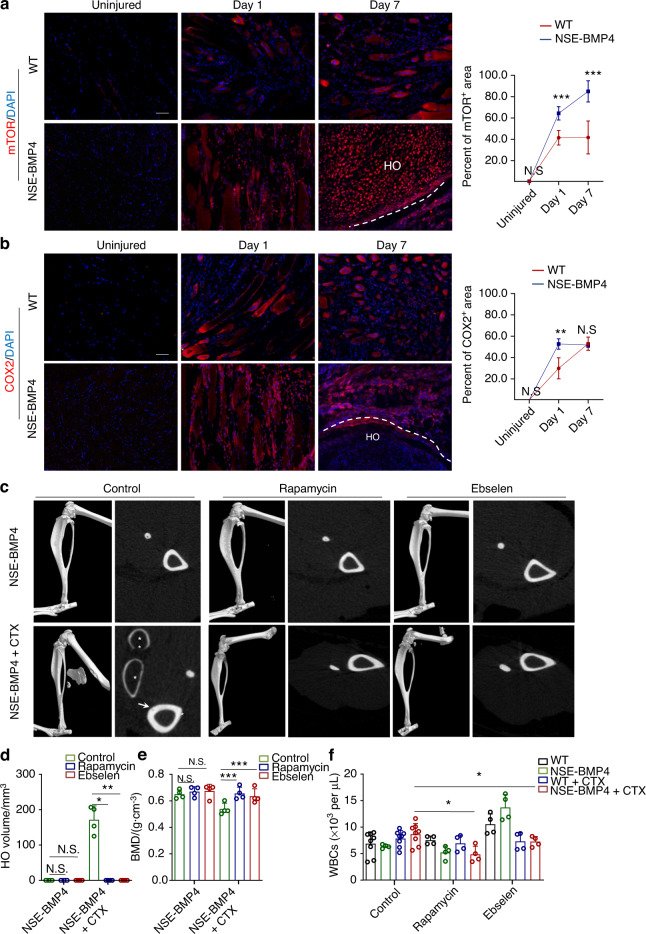
Compounds with anti-inflammatory activity, rapamycin and ebselen, prevent HO and bone loss. **a** IF staining images and statistical analysis of mTOR expression in injured WT and NSE-BMP4 mouse muscle at 1 or 7 dpi. Scale bar, 200 μm. Data are presented as the mean ± s.d. of biological replicates. ^***^P < 0.001, N. S. indicates no significance (unpaired two-tailed *t* test). **b** IF staining images and statistical analysis of COX2 expression in the injured WT and NSE-BMP4 mouse muscle at 1 or 7 dpi. Scale bar, 200 μm. Data are presented as the mean ± s.d. of biological replicates. ^**^P < 0.01, N. S. indicates no significance (unpaired two-tailed *t* test). **c** Representative microCT images of uninjured or injured hindlimbs and a selected transverse section of tibia from the NSE-BMP4 mice with or without drug treatment for two weeks (e.g., rapamycin or ebselen). Arrows indicate bone loss. Asterisks indicate HO. Statistical analysis of the HO volume (**d**) and BMD (**e**) in the uninjured or injured NSE-BMP4 mice following drug treatment (*n* = 3 from two independent experiments). Data are presented as the mean ± s.d. of biological replicates. ^*^P < 0.05, ^**^P < 0.01, ^***^P < 0.001, N. S. indicates no significance (unpaired two-tailed *t* test). **f** Statistical analysis of the numbers of total WBCs in the NSE-BMP4 and WT mice with or without injury following anti-inflammatory drug treatment. Data are presented as the mean ± s.d. of biological replicates. ^*^P < 0.05 (unpaired two-tailed *t* test)

Given that aspects of mammalian target of rapamycin (mTOR) signaling reportedly contribute to the chondrogenesis and osteogenesis responsible for HO development^21–24^, we investigated whether mTOR signaling is involved in the early injury-induced inflammatory stages of HO. We performed immunostaining to examine mTOR expression and found that mTOR was activated upon HO initiation (on postinjury Day 1) and remained activated on postinjury Day 7 (Fig. 3a). Moreover, the mTOR^+^ area in the injured tibial muscle of the NSE-BMP4 mice was higher than that in the WT mice at 1 and 7 dpi. CD3^+^ T cells and CD16/32^+^ macrophages (an inflammatory macrophage subtype) expressed mTOR (Fig. S11a). To evaluate the effect of mTOR inhibition on inflammation and HO, we again induced tibial muscle injury in NSE-BMP4 mice but treated the mice with *i.p*. injections of rapamycin, an inhibitor of the mTOR pathway, once every two days for 2 weeks. MicroCT analysis clearly showed that the development of HO was blocked by rapamycin treatment (Fig. 3c, d). Moreover, this treatment blocked the HO-associated bone loss (Fig. 3e and Fig. S12a) and attenuated the increases in total WBC, lymphocyte and monocyte counts in the injured NSE-BMP4 mice (Fig. 3f and Fig. S12b–e).

Activated immune responses are usually accompanied by a reactive oxygen species (ROS) burst^25^. We therefore used immunostaining to evaluate the expression of the COX2 protein (a marker of ROS and proinflammatory responses^26^) at injured and normal tibia sites. RNA sequencing showed that *Cox2* expression at the injury site of the NSE-BMP4 mice was higher than that at the injury site of the WT mice on Days 1 and 3 post-injury (Fig. S12f). IF staining also confirmed that COX2 was highly expressed in the injured sites of the NSE-BMP4 mice at 1 and 7 dpi but rarely observed in mature HO (Fig. 3a), *i.e*., compared to the WT mice, and a significant increase in COX2^+^ area was observed in the injured tibial muscle of the NSE-BMP4 mice at 1 dpi but not 7 dpi. Additional costaining indicated that COX2 was also expressed in CD3^+^ T cells and CD16/32^+^ M1 macrophages (Fig. S11b). We also conducted experiments in which ebselen was applied on Day 1 post-injury to inhibit ROS production: this treatment blocked HO (Fig. 3c, d) and HO-associated bone loss (Fig. 3e and Fig. S12a). Injury-induced leukocytosis was mildly inhibited in the NSE-BMP4 mice (Fig. 3f and Fig. S12b–e). These results demonstrate that chemically inhibiting early postinjury inflammatory responses, including mTOR- and ROS-mediated pathways, blocks HO pathogenesis.

### A suppressive immune response is a hallmark of BMP4-dependent HO propagation with bone loss

**Fig. 4:**
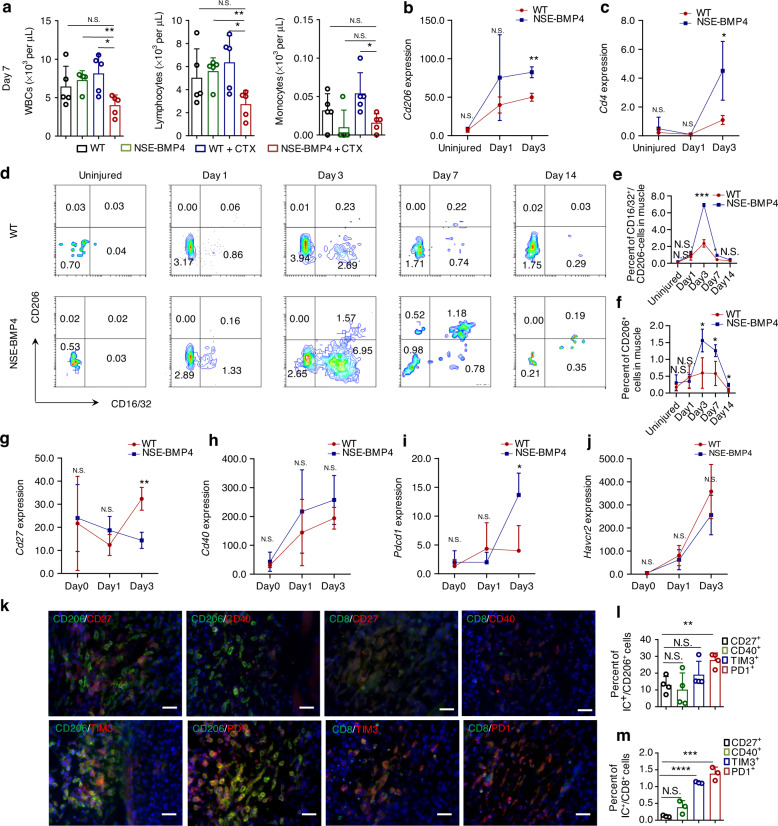
A suppressive immune response is a hallmark of HO propagation with bone loss. **a** Statistical analysis of peripheral WBCs, lymphocytes and monocytes in the WT and NSE-BMP4 mice at 7 dpi (*n* = 5 from three independent experiments). Data are presented as the mean ± s.d. of biological replicates. ^*^P < 0.05, ^**^P < 0.01, N.S. indicates no significance (unpaired two-tailed *t* test). Statis*t*ical analysis of the expression of *Cd206* (**b**) and *Cd4* (**c**) in the WT and NSE-BMP4 mouse muscle via an RNA-sequencing assay (*n* = 3 per group). Data are presented as the mean ± s.d. of biological replicates. ^*^P < 0.05, ^**^P < 0.01. N.S. indicates no significance. **d** Representative flow cytometry images of macrophage polarization in uninjured or injured muscle from the WT and NSE-BMP4 mice. Statistical analysis of the population of M1 macrophages (**e**) and M2 macrophages (**f**) in uninjured or injured muscle from the WT and NSE-BMP4 mice (*n* = 3 per group). Data are presented as the mean ± s.d. of biological replicates. ^*^P < 0.05, ^***^P < 0.001. N.S. indicates no significance (unpaired two-tailed *t* test). **g–j**, Statistical analysis of the expression of *Cd27*, *Cd40*, *Pdcd1* and *Havcr2* in injured muscle from the WT and NSE-BMP4 mice at different postinjury times (*n* = 3 per group). Data are presented as the mean ± s.d. of biological replicates. ^*^P < 0.05, ^**^P < 0.01. N.S. indicates no significance (unpaired two-tailed *t* test). **k** At 7 dpi, immunostaining showed colocalization of CD206 or CD8 and immune checkpoint molecules in HO lesions. **l, m** Statistical analysis of the population of CD206^+^ or CD8^+^ cells expressing immune checkpoint molecules (*n* = 3 per group, *n* = 3 ROI/mouse). Data are presented as the mean ± s.d. of biological replicates. ^**^P < 0.01, ^***^P < 0.001, ^****^P < 0.000 1. N.S. indicates no significance (unpaired two-tailed *t* test)

**Fig. 5:**
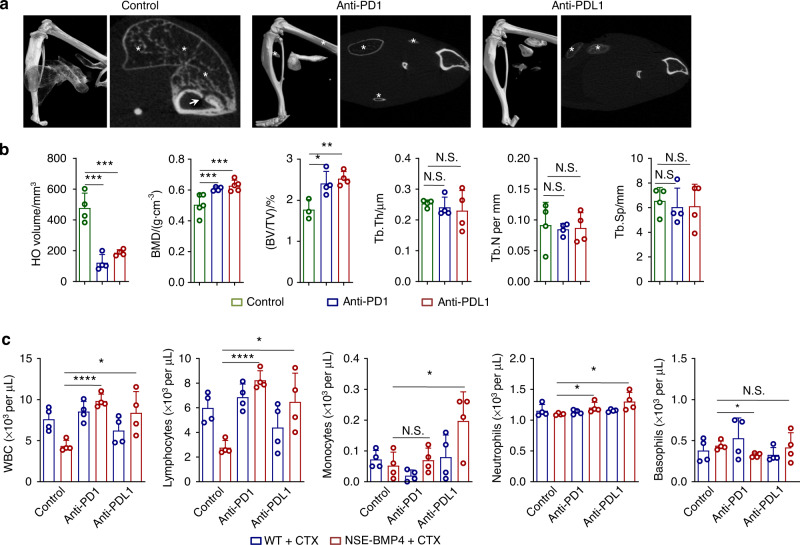
Neutralization of PD1/PDL1 alleviates HO progression and bone loss. **a** Representative microCT images of injured hindlimbs and a selected transverse section of tibia in the NSE-BMP4 mice treated with or without immune checkpoint blockade for two weeks. Arrows indicate bone mass loss. Asterisks indicate HO. **b** Statistical analysis of the HO volume, BMD and other bone parameters in the injured NSE-BMP4 mice following treatment with anti-PD1 or anti-PDL1 Abs (*n* = 4-5 per group). Data are presented as the mean ± s.d. of biological replicates. ^*^P < 0.05, ^***^P < 0.001. N.S. indicates no significance (unpaired two-tailed *t* test). **c** Statistical analysis of the numbers of peripheral WBCs, lymphocytes, monocytes, neutrophils and basophils in the injured WT and NSE-BMP4 mice following PD1 or PDL1 blockade (*n* = 3 per group). Data are presented as the mean ± s.d. of biological replicates. ^*^P < 0.05, ^****^P < 0.000 1. N.S. indicates no significance (unpaired two-tailed *t* test)

As noted above, the altered tibial BMD and proliferative osteoclast phenotypes (including cortical and femoral osteoclasts) of the injured NSE-BMP4 mice were evident on postinjury Days 7 and 14 but not on postinjury Day 1 (Fig. S2a, b, Fig. S4a, b and Fig. 5a, b). Additionally, based on our findings that the expression of *Cd4* and *Cd206* was significantly upregulated by postinjury Day 3 (Fig. S13a) and that these genes function in adaptive and innate immunosuppression^27,28^, we speculated that suppression of immune responses may occur in HO lesions. If so, such immunosuppression may contribute to HO-associated bone loss. Pursuing this, we sampled blood to monitor immune responses as mature HO developed post-injury. Surprisingly, we detected substantially reduced peripheral WBC levels in the injured NSE-BMP4 mice compared to the injured WT controls from Day 7 onward—a point when definite HO is well established in NSE-BMP4 mice (Fig. 4a and Fig. S13b). The peripheral WBC levels in the injured NSE-BMP4 mice remained decreased at 14 dpi (Fig. S13c).

Moreover, we investigated potential trends in immunosuppression specific to HO lesions in our aforementioned RNA-sequencing dataset for cells sampled from HO lesions. Our RNA-sequencing data for postinjury Day 3 showed no significant differences in the expression of *Fcgr3* (a marker of M1 macrophages) between HO lesions from the NSE-BMP4 mice and injured tibia sites of the WT mice (Fig. S13d). However, the expression of *Cd206* (a marker of M2 macrophages) was higher in the HO lesions than in the injured sites of tibial muscle of the WT mice (Fig. 4b). *Cd4* expression in the HO lesions was persistently increased beginning on Day 3 post-injury compared to that in the corresponding injured tibia sites of the WT mice (Fig. 4c). However, the expression of *Cd8a*, which represents an active adaptive immune response, did not significantly change from 1 to 7 dpi in the HO lesions (Fig. S13e). Flow cytometry analysis further showed that the population of CD16/32 ^+^ /CD206^−^ M1 macrophages at the injury site in the NSE-BMP4 mice peaked on postinjury Day 3 but drastically decreased on postinjury Day 7 (i.e., almost the same as that at 1 dpi) (Fig. 4d, e and Fig. S14a–c). In contrast, the population of CD206^+^ M2 macrophages increased in the HO lesions at 3, 7 and 14 dpi compared to that at 1 dpi (Fig. 4d, f and Fig. S14a–c). More importantly, the population of CD206^+^ M2 macrophages in the HO lesions was significantly higher than that in the injured tibial sites of the WT mice from 3 dpi onward (Fig. 4d, f). Additionally, the difference in the myeloid cell population between the HO lesions and injured sites in WT muscle did not significantly differ (Fig. S15a, b). IF staining showed that the population of CD4^+^ T cells in the HO lesions progressively increased with increasing postinjury time, whereas no changes were observed in the number of CD8a^+^ T cells (Fig. S15c–f). Collectively, these findings clearly indicate that after a strong immune response upon initial injury, the progression of HO lesions is accompanied by immunosuppression that dampens both innate and adaptive immune functions.

### A suppressive immune context at the injury site promotes HO propagation and bone loss via PD1 dysregulation

The suppressive immune phenotypes we observed during the HO propagation stage appear quite similar to the known tumor-promoting immunosuppressive phenotypes of many cancers^29^. This similarity prompted us to hypothesize that HO-initiating cells may undergo proliferation without facing immune surveillance responses. Indeed, PDGFRα^+^ HO-initiating cells expressed PDL1^30,31^ (Fig. S13f). In addition, we examined the expression of inhibitory and stimulatory immune checkpoint molecules, such as *Cd27*, *Cd40*, *Pdcd1* and *Havcr2* (Fig. 4g–j). Only *Pdcd1* expression in the HO lesions was increased significantly compared to that in the injured sites of the WT mice (Fig. 4i). Instead, *Cd27* was markedly decreased in the HO lesions compared with the injured sites of the WT mice (Fig. 4g). Moreover, M2 macrophages were largely PD1^+^ or TIM3^+^ but not CD27^+^ or CD40^+^ (Fig. 4k, l). Consistently, CD8^+^ T cells also expressed PD1 or TIM3, markers of terminally exhausted T-cell populations^32^ (Fig. 4k, m). We next tested whether this immune dysregulation during HO propagation is the cause of the observed bone loss. We repeated the injury procedure. On postinjury Day 7, we intravenously injected anti-PD1/PDL1 neutralizing antibodies into NSE-BMP4 mice (daily for 2 weeks). PD1/PDL1 blockade significantly slowed HO progression (Fig. 5a, b). MicroCT data showed that compared to the mice in the control group (injured NSE-BMP4 mice without anti-PD1/PDL1 treatment), the NSE-BMP4 mice in the anti-PD1/PDL1 neutralizing Ab-treated group showed an enhanced BMD and BV/TV; no changes were observed for Tb. Th, BS/BV, Tb. N, or Tb. Sp (Fig. 5b). Moreover, neutralization of PD1/PDL1 stimulated WBC generation, especially lymphocyte generation, indicating that a deficiency in adaptive immunity promoted HO progression (Fig. 5c). Collectively, these results support the idea that an immunosuppressive microenvironment promotes HO progression and HO-associated bone loss.

### FetA is frequently detected in HO lesions but rarely observed in HO-adjacent normal bone

How does the suppressive immune response in HO contribute to the observed bone mass loss? Since the immunosuppressive microenvironment begins to form on postinjury Day 3 (evident from CD4 and CD206 expression in HO lesions), we hypothesized that i) suppressive immune responses in HO lesions beginning on postinjury Day 3 may promote osteoclast generation, potentially from the differentiation of lesion-resident macrophages, and ii) newly generated osteoclasts may have the capacity to migrate from injury sites to the bone responsible for bone loss. We first assessed the expression of *Ctsk*, an osteoclast marker, but the RNA-sequencing data showed no difference between the injured NSE-BMP4 and WT mice on postinjury Day 1 or 3 (Fig. S16a). Tartrate-resistant acid phosphatase (TRAP) assays and IF staining confirmed that TRAP^+^ cells accumulated in the region that was abundant in macrophages (Fig. S16b, d). These results indicated that no osteoclasts were generated in the injured tibial muscle of the NSE-BMP4 mice at 1 or 3 dpi. The migration of osteoclasts to the bone was also not found.

We next hypothesized that cytokines produced on postinjury Day 3 in HO lesions may promote immunosuppression and bone loss. Since activin A (ActA) has been proposed to be a regulatory factor in immune responses and chondrogenic differentiation of MSCs in HO^33,34^, we used ELISAs to measure the expression of ActA at different time points during HO progression. ActA was found to be dynamically dysregulated in this model, *i.e*., ActA levels in the serum of the injured NSE-BMP4 mice were transiently increased on Days 1 and 14 (Fig. S17a); however, on Days 3 and 7, ActA expression in the NSE-BMP4 mice was actually downregulated compared to that in the injured WT mice (Fig. S17a). Moreover, when we treated the injured WT and NSE-BMP4 mice with the mTOR inhibitor rapamycin for 2 weeks, the rapamycin-treated NSE-BMP4 mice still had high ActA levels, even though these animals exhibited the same blockade in HO and attenuated leukocytosis that we observed earlier (Fig. S17b). This apparently paradoxical result would make sense if the dysregulation of ActA is unrelated to HO pathogenesis in this disease model; minimally, it seems clear that ActA does not obviously impact HO or bone loss in this model, which is consistent with a previous study^35^. Notably, we did not detect significant differences in ActA expression in the blood between patients with injury-induced HO and healthy controls or hip fracture patients (Fig. S17c).

**Fig. 6:**
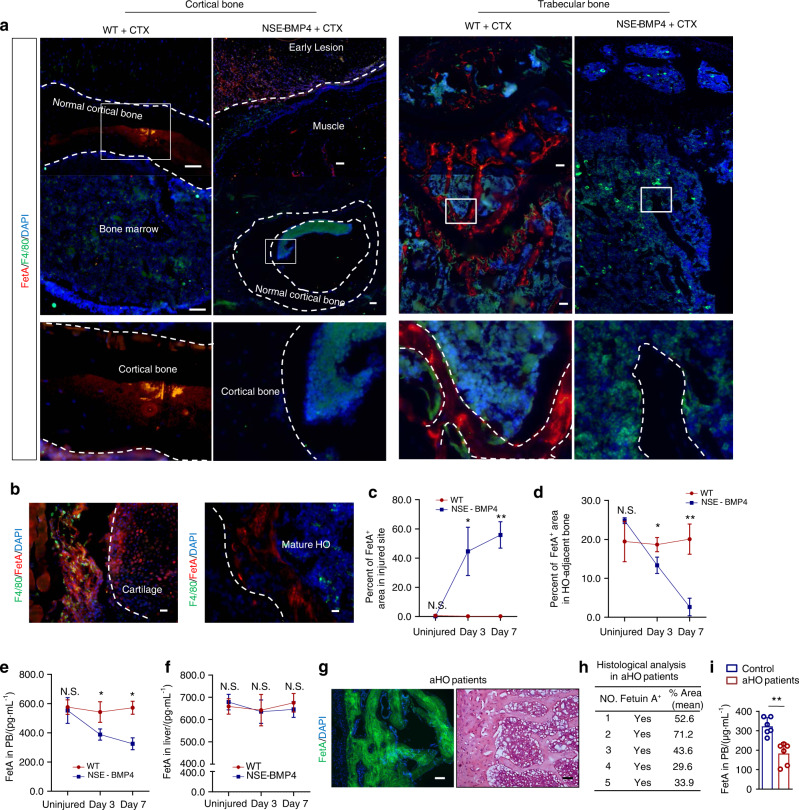
FetA is detected frequently in HO lesions but rarely in normal bone. **a** Immunostaining images of FetA in the cortical and trabecular bone of the injured WT mice and HO lesions and adjacent bone. Scale bar, 200 μm. **b** Immunostaining images of FetA^+^ and F4/80^+^ cells at the cartilage-developing stage and mature HO stage. Scale bar, 200 μm. **c** Statistical analysis of the FetA^+^ area in uninjured or injured muscle from the WT and NSE-BMP4 mice at different postinjury times (*n* = 3, *n* = 3 ROI/mouse). Data are presented as the mean ± s.d. of biological replicates. ^*^P < 0.05, ^**^P < 0.01 (unpaired two-tailed *t* test). **d** Statistical analysis of the FetA^+^ area in normal bone (from the injured WT mice) and HO-adjacent bone (from the injured NSE-BMP4 mice) at different postinjury times (*n* = 3 ROI/mouse). Data are presented as the mean ± s.d. of biological replicates. ^*^P < 0.05, ^**^P < 0.01 (unpaired two-tailed *t* test). Statistical analysis of FetA expression in the peripheral blood (*n* = 5) (**e**) and liver (*n* = 5) (**f**) of the uninjured or injured WT and NSE-BMP4 mice by ELISAs. Data are presented as the mean ± s.d. of biological replicates. ^*^P < 0.05, N.S. indicates no significance (unpaired two-tailed *t* test). **g** Representative FetA immunostaining and HE s*t*aining images of HO samples from patients with aHO. Scale bar, 200 μm. **h** Histological analysis of the FetA^+^ region in the mature HO lesions of patients with aHO (*n* = 5 donors). Data are presented as the mean ± s.d. of biological replicates. **i** Statistical analysis of FetA expression in the blood of patients with aHO and healthy controls (*n* = 5 donors). Data are presented as the mean ± s.d. of biological replicates. ^**^P < 0.01 (unpaired two-tailed *t* test)

If not ActA, as was anticipated, what underlies this immunosuppressive phenotype and the subsequent bone loss? *Il-6* and *Tnfsf11* have been previously implicated in bone loss. The levels of both of these molecules were increased in HO lesions (Fig. S17d) but were not different in the bone between the model mice and the WT control mice (Fig. S17e). Fetuin-A (FetA), also known as Alpha-2-HS-glycoprotein (*Ahsg*), is mainly expressed and secreted by the liver and adipose tissue and has been reported to be involved in immune regulation^36^, valve calcification^37^ and osteoporosis^38^. Therefore, we next tested FetA expression in HO lesions and adjacent bone. Interestingly, at 3 dpi, FetA was expressed in the injured site of tibial muscle of the NSE-BMP4 mice but was rarely detected in bone (including cortical and trabecular bone) adjacent to HO lesions. Instead, the bone matrix of the WT mice showed a strong signal for FetA (Fig. 6a). Further costaining between FetA and F4/80 indicated that macrophages expressed FetA at 3 dpi and consequently localized to the HO matrix at 7 dpi (Fig. 6b). In addition, at 7 dpi, FetA^+^ cells expressed SOX9 (a marker of chondrocytes) but not endomucin (a marker of vascular endothelial cells), CD3 or RUNX2, suggesting another role of FetA in chondrocytes other than its immunoregulatory function at 3 dpi (Fig. S18a–d). Statistical analysis of the FetA^+^ area in injured muscle revealed that the injured NSE-BMP4 mice exhibited enhanced FetA expression in the injured site of tibial muscle compared with the WT mice (Fig. 6c). However, FetA expression in HO-adjacent bone of the NSE-BMP4 mice was significantly decreased compared with that in the WT mice (Fig. 6d). Moreover, ELISAs revealed significantly decreased FetA levels in the blood of the injured NSE-BMP4 mice compared to that of the injured WT mice at 7 dpi (Fig. 6e). Notably, the FetA level was not altered in the liver (a site of FetA production and secretion into the blood) of the injured NSE-BMP4 mice at 7 dpi (Fig. 6f). To explore any clinical relevance of this finding, we collected HO samples from 5 patients with femoral or tibial injury-induced HO. An immunostaining assay showed that FetA was indeed highly expressed in mature HO (Fig. 6g, h). We also confirmed that FetA levels were decreased in the circulation of patients with aHO (Fig. 6i). In summary, these results suggest that FetA may regulate HO with bone loss.

### FetA acts as an immunomodulator to enhance PD1 expression, and consumption of FetA prevents BMP4-dependent HO and associated bone loss

**Fig. 7:**
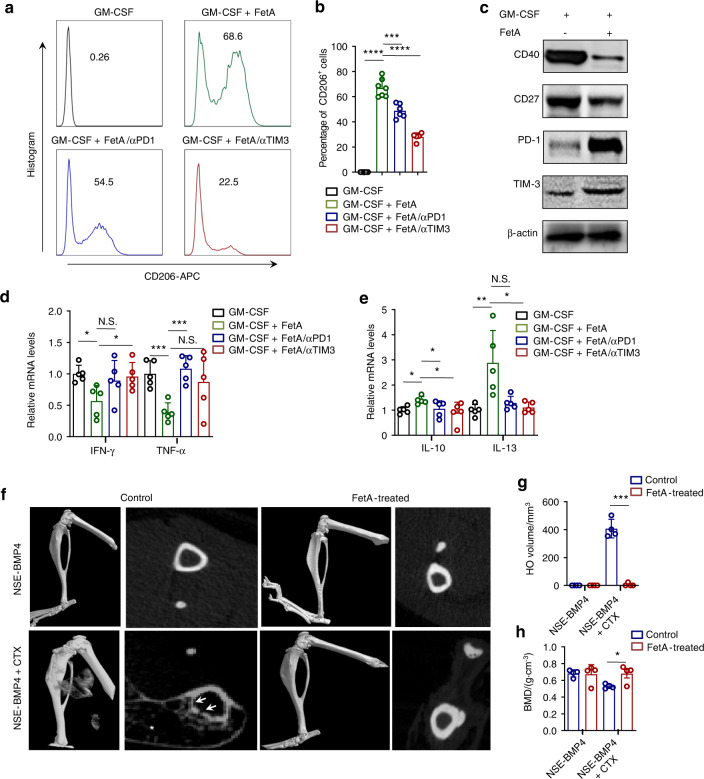
FetA acts as an immunomodulator to enhance PD1 expression. **a** Flow cytometry analysis of the population of CD206^+^ M2 macrophages after FetA and anti-IC molecule Ab treatment. **b** Statistical analysis of the percentage of CD206^+^ macrophages after FetA and/or anti-IC molecule Ab treatment. Data are presented as the mean ± s.d. of biological replicates. ^***^P < 0.001, ^****^P < 0.000 1 (unpaired two-tailed *t* test). **c** Western blot analysis of immune checkpoint molecule expression in the GM-CSF-induced M1 macrophages with or without FetA treatment. Statistical analysis of proinflammatory (**d**) and anti-inflammatory cytokine (**e**) expression in macrophages after FetA and anti-IC molecule Ab treatment. Data are presented as the mean ± s.d. of biological replicates. ^*^P < 0.05, ^**^P < 0.01, ^***^P < 0.001, N.S. indicates no significance (unpaired two-tailed *t* test). **f** Representative microCT images of injured hindlimbs and a selected transverse section of tibia in the NSE-BMP4 mice treated with or without FetA for two weeks. Statistical analysis of the HO volume (**g**) and BMD (**h**) in the NSE-BMP4 mice (*n* = 3-4 per group) treated with or without FetA. Data are presented as the mean ± s.d. of biological replicates. ^*^P < 0.05, ^***^P < 0.001 (unpaired two-tailed *t* test)

FetA is known to stabilize mineral substances and stimulate immune responses^37,39^. However, FetA was already enriched in HO lesions on Day 3 post-injury in our model, suggesting that FetA might be an immunomodulatory protein that promotes HO and bone loss. Given our observations that the immunosuppressive microenvironment is controlled by immune checkpoints (ICs), we first investigated whether FetA mediates the dysregulation of ICs. Bone marrow-derived macrophages (BMDMs) were generated to investigate the potential functional impacts of FetA on ICs. BMDMs were treated with recombinant FetA protein in 7-day cultures (macrophages were cultured with serum replacement). As expected, FetA induced morphological transformation from fried egg–like (M1) to spindle-like (M2) macrophages (Fig. S19a). Furthermore, FCM and qPCR assays indicated that FetA promoted M1 to M2 polarization in BMDMs (Fig. 7a, b, d, e and Fig. S19b). Additionally, PD1 and TIM3 expression but not CD27 or CD40 expression was enhanced in the FetA-exposed BMDMs (Fig. 7c). Furthermore, anti-PD1/TIM3 neutralizing antibodies reversed this phenotypic enhancement (Fig. 7a, b, d, e). Our aforementioned immunostaining indicated that in the injured NSE-BMP4 mice, the number of osteoclasts was abnormally increased in the bone adjacent to HO (Fig. S4a, b). Moreover, stimulatory IC molecules, including CD27 and CD40, were both activated in osteoclasts at the injury site in the NSE-BMP4 mice compared to the WT mice (Fig. S19c). No positive signals for inhibitory ICs were detected (data not shown). More importantly, when we treated the NSE-BMP4 mice with recombinant FetA 3 days post-injury, the expression of PD1 increased, but macrophage infiltration decreased (Fig. S19d). When FetA treatment lasted for 2 weeks, HO and associated bone loss could be prevented (Fig. 7f-h and Fig. S19e). Taken together, these results suggest that FetA may act as an immunomodulator to stimulate PD1. In addition, intervention with FetA inhibits hyperinflammation and prevents BMP4-dependent HO and associated bone mass loss.

## Discussion

Our work established a link between HO pathogenesis and its effect on bone structure and provided potential mechanisms linking HO pathogenesis with bone mass loss. We showed that bone mass loss is a pathological phenotype of injury-induced and BMP4-dependent HO and demonstrated that rather than the early inflammatory response (1 day after injury), later (7 days after injury) suppressive immune response-mediated HO propagation leads to local bone loss. In particular, we identified FetA as an immunomodulator promoting PD1 expression, which suggests that FetA is a potential option to prevent HO or a therapeutic target for the treatment of HO and associated bone loss.

Bone loss is initiated by osteoclast activation. Macrophages are an important cellular component in HO formation^12,17^. We found that monocyte counts were abnormally increased in the blood of the NSE-BMP4 mice 1 day after injury, and monocytes are known to generate macrophages and osteoclasts^20^. At 1 day post-tibial muscle injury, monocyte numbers in the NSE-BMP4 mice were increased, and these cells possibly contributed to bone loss. However, no osteoclast activation was found in the bone of the NSE-BMP4 mice until 7 days after injury. Osteoclasts can be activated after inflammatory cytokine induction for 4 days in vitro^40,41^. In vivo renewal of osteoclasts requires only 24 h^42^. Thus, direct regulation of osteoclasts by monocyte differentiation seems unlikely.

Innate and adaptive immune cells contribute to HO and bone diseases^9,11,12,43,44^. However, the roles of these immune cells linked with HO and bone loss remain unclear. Inflammation is the central cause of bone loss. Did higher number of immune cells in HO model mouse blood represent an inflammatory response? Leukocytosis is known to be a common sign of infection/injury, but a potential problem of this approach is that the peripheral WBC count can also be modified by many “nonspecific” stimuli, including surgery, exercise^45^, trauma^46^, stress, obesity, aging, pregnancy and certain medications^47,48^; however, we argue that if “nonspecific” stimuli are well controlled, such as in our animal study, the peripheral WBC count should be a valuable parameter to gauge the immune response. Similarly, local WBC infiltration also does not seem to be a very specific parameter; however, it seems that local leukocyte extravasation (also known as diapedesis) is controlled more stringently. However, it is worth mentioning that “infiltration” itself is not necessarily a pathological process or a definite way to gauge the immune response. For example, when monocytes develop into tissue macrophages, monocytes can undergo the extravasation process even in the absence of infection or tissue damage.

The inflammatory response is actually a systemic process that includes immune mediator circulation and accumulation at the injury site. Characterization of the key factors that reflect this inflammatory response is extremely important to explain the pathogenesis of HO with bone loss. In the present study, we found that an early acute inflammatory response triggered HO but not bone loss. We then found that from Day 7 onward, the number of circulating WBCs, especially lymphocytes and monocytes, was drastically decreased in the injured HO model mice compared to the injured WT mice. This decrease in the number of lymphocytes and monocytes in the injured NSE-BMP4 mice might indicate that immune dysfunction is closely associated with HO pathogenesis. Our previous study showed that inhibitory immune checkpoints, which determine immune suppression, are essential to HO formation^13^. These results led us to consider whether immunosuppression is the cause of HO and associated bone loss. The decrease in leukocytosis normally takes longer, especially when an ongoing inflammatory response still exists^46^. The specific implications from the lag in leukocytosis compared to the overall immune status observed in our study might indicate overactive early and depressed late leukocytosis. Accordingly, it is possible that, at some time points in the middle of HO development (for example, Day 3 after injury), the body is already in an immunodeficient state despite a high WBC count. Indeed, local immune cells in the NSE-BMP4 mice expressed immunosuppressive markers at Day 3 post-injury, such as CD4 and CD206. These suppressive immune cells were abundantly PD1^+^. We further performed a rescue assay involving treatment with immunosuppressants or immune activators; the results illustrated that the immunosuppressive microenvironment was the central mechanism driving the pathogenesis of HO with bone loss.

In biomineralization, FetA is a potent inhibitor of ectopic mineralization, *i.e*., monomeric FetA binds to and transports small clusters of calcium and phosphate to prevent calcification in soft tissue, such as blood vessels^37^. Interestingly, during HO formation, FetA was not only enriched in soft tissue but also displayed decreased levels in the circulation and bone. This finding suggested that systemic FetA might egress to HO lesions, including the region where immune cells aggregate (especially F4/80^+^ cells) and the HO matrix. We speculated that FetA could stimulate chemokine signaling, leading to recruitment of immune cells to HO lesions. In fact, FetA was reported to promote macrophage migration^49^. Therefore, chemotactic signaling could be important for FetA-induced macrophage recruitment. Chemokine signaling was reported to participate in normal bone remodeling, partly through the osteoblast-derived CXCL9 effect on angiogenesis in bone^50^. Additionally, SDF1 can recruit MSCs, which are HO-initiating cells^31,51^. Thus, FetA may regulate chemokine signaling, especially signaling via the SDF1 and CXCL9 pathways, contributing to HO formation.

Additionally, FetA accumulation may further regulate the immune response to promote HO formation. FetA exhibits two contradictory effects on the immune system in the context of pathological models^52^; on the one hand, FetA is divergently regulated by different proinflammatory mediators and functions as a positive or negative acute phase protein (APP) in injury and infection. This phenomenon not only facilitates the anti-inflammatory actions of cationic polyamines (e.g., spermine) but also directly inhibits damage-associated molecular pattern (DAMP)-induced high mobility group box-1 (HMGB1) release by innate immune cells^53^. On the other hand, plasma FetA triggers inflammatory changes through the activation of Toll-like receptor 4 (TLR-4) pathways^36,39^. Our model confirmed that FetA could stimulate PD1 and facilitate M2 macrophage polarization, exhibiting an anti-inflammatory effect. Moreover, we demonstrated that this abnormal polarization is mediated by IC dysregulation. We have previously shown that IC dysregulation facilitates HO formation^13^. In this study, we further demonstrated that FetA could stimulate IC molecules to decrease macrophage infiltration or promote macrophage polarization and osteoclast activation. Our group and others have revealed that macrophage deletion could inhibit HO^12,54^. Recently, multiple studies revealed that different types of macrophages play roles along with different stages of HO^55,56^. M1 macrophages secrete inflammatory cytokines and trigger HO-initiating cell proliferation, which could be involved in the inflammatory stage of HO^57^. M2 macrophages might promote immunosuppression and thus promote HO-initiating cell differentiation into HO^58^. Levi et al. showed that the percentage of CD206^+^ macrophages (M2-like) and *Tgfb1* expression were increased at 3 dpi but not 1 dpi, which is consistent with our study^44^. Thus, suppression of inflammation at the early stage or reversal of immunosuppression from 3 dpi onward can inhibit HO. We chose intervention with FetA at the early inflammatory stage to promote infiltrated M1 macrophage polarization into M2 macrophages and subsequently prevent hyperinflammation. Inhibition of inflammation prevents HO-initiating cell proliferation. Therefore, FetA-induced M2 macrophages at this time promote muscle repair^59^ but not HO formation. Hence, we believe that FetA can modulate the inflammatory response to prevent HO formation. Osteoclasts originate from macrophages/monocytes, so it is important to evaluate the link between ICs and macrophage polarization and/or osteoclast activation in bone marrow-derived macrophages. To the best of our knowledge, these results are the first to demonstrate the relationship between ICs and FetA. More importantly, we demonstrated that FetA stimulates PD1 to promote immunosuppression. FetA supplementation at an early stage could prevent HO.

In addition to regulating the immune response at 3 dpi, FetA could further regulate chondrocyte development, as SOX9^+^ chondrocytes expressed FetA at 7 dpi. Although FetA was reported to inhibit mineralization in vitro, FetA-deficient mice showed normal bone material properties^60^. However, mice lacking FetA were shorter than wild-type mice and had a higher mineral content in the growth plate, suggesting that inhibition of FetA promotes premature mineralization of the growth plate^60^. FetA^+^ chondrocytes in our HO model mice could control the homeostasis of lesional chondrocyte mineralization and eventually promote heterotopic endochondral bone formation. Notably, FetA was not expressed by RUNX2^+^ osteoblasts but was distributed around osteoblasts. FetA may bind calcium phosphate in the extracellular matrix (ECM) region^37^, thus promoting HO stabilization. Thus, FetA accumulation in HO, at 7 dpi, may regulate chondrocyte maturation and subsequently heterotopic bone formation.

Although we showed that HO-associated bone mass loss occurred in BMP4-dependent mice and patients with traumatic HO, the relationship between HO and bone mass loss still needs to be proven by further investigations in a traumatic HO mouse model and a fibrodysplasia ossificans progressiva (FOP) mouse model. Additionally, FetA was characterized as a decoy receptor of transforming growth factor beta (TGF-β)/BMP, suggesting that FetA could be useful to inhibit and/or prevent TGF-β/BMP-induced diseases, including heterotopic ossification^61–64^. However, the mechanistic role of FetA in traumatic HO and FOP mouse models should be confirmed, since other cytokines, for example TGF-β, as well as BMP4 activation, participate in HO pathogenesis. Together, we revealed that FetA mediated HO formation and associated bone mass loss in BMP4-dependent HO. Moreover, our findings help elucidate the HO pathogenesis and support the therapeutic potential of targeting FetA as an effective approach to prevent HO.

## Methods

### Mice and patient samples

NSE-BMP4 transgenic mice were a gift from Dr Kan Lixin (Northwestern University, USA). As described previously, the *Nse-Bmp4* transgene was constructed by cloning a 1 246-bp fragment containing the murine *Bmp4* cDNA downstream of the rat neuron-specific enolase (*Nse*) promoter and upstream of an SV40 polyadenylation signal. All experimental procedures involving mice were carried out as prescribed by the National Guidelines for Animal Usage in Research (China) and were approved by the Ethics Committee of Anhui Medical University (reference: LLSC20140042; Hefei, China). Peripheral blood samples and HO tissues of patients with HO were obtained from the First Affiliated Hospital of the University of Science and Technology of China (Hefei, China). The experiments involving human subjects were reviewed and approved by the Institutional Review Board of the University of Science and Technology of China (approval number: P-023; Hefei, China). The clinical characteristics of all patients included in the present study are shown in Tables S3–5.

### Injury-induced and BMP4-dependent HO model

HO was induced by intramuscular injection of cardiotoxin (217503, Sigma) into NSE-BMP4 mice according to a protocol published in previous reports. Briefly, 100 μL of cardiotoxin (8 μg·kg^−1^) in PBS was injected into the tibial muscle of NSE-BMP4 mice (6–8 weeks old). Care was taken to avoid incidental contact with the tibia. Injections were performed while the mice were under isoflurane anesthesia.

### MicroCT

Mouse hindlimbs were harvested and imaged after injury at different time points. For quantitative measurement of the HO volume and bone parameters, microCT (Skyscan 1176, Bruker) was used with the setting parameters of 180° rotation, a constant 90-kV voltage and a voxel size of 72 μm. Then, 3D images were reconstructed with SkyScan software. The HO region was first outlined by the ROI module and then quantified by individual 3D object analysis. For tibial and femoral bone parameter analyses, an ROI module was used to outline the entire tibia and femur separately. Individual 3D object analysis was used to calculate the bone parameters, such as the bone mineral density (BMD), the ratio of bone volume to tissue volume (BV/TV), the ratio of bone surface to bone volume (BS/BV), the number of trabeculae (Tb. N), trabecular separation (Tb. Sp) and trabecular thickness (Tb. Th).

### Histology analysis

WT and NSE-BMP4 mouse hindlimbs with or without injury were harvested at different time points and then subjected to the indicated staining. Briefly, hindlimbs were fixed at 4 °C in 4% paraformaldehyde or 10% neutral-buffered formalin. The samples were then decalcified in 20% (m/v) EDTA solution for 4–6 weeks at 4 °C until they were manually deformable. Bones were paraffin-embedded, and 10 μm sections were cut and stored at −20 °C. Paraffin sections were selected for dewaxing, and then, HE staining (HE staining Kit, G1120, Solarbio), safranin O staining (Modified safranine O-fast green FCF cartilage staining kit, G1371, Solarbio), Masson trichrome staining (Masson trichrome staining kit, BP-DL023, Sbjbio) and TRAP staining (TRAP staining Kit, G1492, Solarbio) assays were performed according to the manufacturer’s instructions.

### RNA sequencing and analysis

Tibial muscle from NSE-BMP4 and WT mice was collected at different time points (Fig. 2b). Total RNA was extracted using QIAzol lysis reagent (Qiagen) according to the manufacturer’s instructions. Sequencing libraries were generated using the NEBNext^®^ Ultra^™^ RNA Library Prep Kit for Illumina^®^ (NEB, USA) following the manufacturer’s recommendations, and index codes were added to attribute sequences to each sample. Clustering of the index-coded samples was performed on the cBot Cluster Generation System using the HiSeq 4000 PE Cluster Kit (Illumina) according to the manufacturer’s instructions. After cluster generation, the library preparations were sequenced on an Illumina HiSeq 4000 platform, and 150 bp paired-end reads were generated. For data analysis, raw data were assessed by quality control, and clean data were output for quantification of gene expression. For samples without biological replicates, differential expression analysis of two conditions was performed using the DEGseq R package (1.28.0). A *P* value <0.05 and fold change ≥2 were set as the thresholds for significance. GO and KEGG enrichment analyses were used to further analyze the DEGs through the DAVID database and KOBAS V3.0 software, respectively.

### RNA isolation and qPCR

TRIzol and the RevertAid First Strand cDNA Synthesis Kit (1622, Thermo) were used to extract total RNA and synthesize first-strand cDNA from lesional tissues and macrophages following the manufacturer’s instructions. The expression of various genes was quantified by real-time PCR mixture assays (TaKaRa, China). β-actin was used as the internal control. All primer sequences are listed in Table S3.

### Immunofluorescence assays

Immunostaining for different markers was performed as previously described^12^. Briefly, sections were prefixed with 4% paraformaldehyde in PBS. Nonspecific binding was blocked with 10% normal goat serum diluted in 1% bovine serum albumin (V900933, Sigma) and 0.25% Triton X-100 (X100, Sigma) for 1 h at room temperature. The sections were then incubated with primary antibodies diluted with 1% BSA + 0.25% Triton X-100 at 4 °C overnight (the primary antibodies used in this study are listed in Table S4). After washing, the sections were incubated with appropriate secondary antibodies (Alexa Fluor 488- or Alexa Fluor 594-conjugated antibodies) diluted with 1% BSA + 0.25% Triton X-100 in the dark at room temperature for 2 h and counterstained with 4,6-diamidino-2-phenylindole (1:4 000). All fluorescence microscopy images were acquired using a Zeiss Axio Observer (Carl Zeiss).

### Immunosuppressant treatment or IC blockade in vivo

NSE-BMP4 mice (*n* = 4) were treated with rapamycin (553210, 5 mg·kg^−1^, Sigma) or ebselen (70530, 1 mg·kg^−1^, Cayman) thrice per week for 2 weeks through i.p. injection beginning 1 Day p.i., and control NSE-BMP4 mice (*n* = 4) were treated with an equal volume of vehicle (PBS) administered the same way. Regarding IC blockade, 1 week after target limb injury, we treated the injured NSE-BMP4 mice with neutralizing antibodies specific for inhibitory IC molecules, including PD1 (BP0273, at 10, 50 or 250 g per injection, BioXCell) and PD-L1 (BP0101, at 24, 120 or 600 g per injection, BioXCell), via tail vein injection, and the 1st injection was performed 1 day after injury. All neutralizing antibodies were administered every two days for 2 weeks. The readouts of these treatments included a) the numbers of peripheral WBCs and their subpopulations at different time points and b) microCT parameters (for HO volume quantification and normal bone analysis).

### TRAP activity

After sacrifice, target tissues were collected from mice to generate frozen sections. A TRAP kit (P0332, Beyotime) was used to test TRAP activity. Briefly, a chromogenic substrate (200 μL) and a tartaric acid solution (25 μL) were mixed and added to target lesions. After 30 min of incubation at 37 °C, 250 μL of stop solution was used to terminate the reaction. Positive signals (black) were visualized with a Zeiss Axio Observer (Carl Zeiss).

### Enzyme-linked immunosorbent assay (ELISA)

A Quantikine ELISA kit (DAC00B, R&D Systems) was used for serum Activin A detection according to the manufacturer’s instructions. The expression level of FetA in the serum of mice or patients was also examined with ELISA kits (Cat# EHAHSG and Cat# EM31RB, Thermo).

### Western blot analysis

Lesional tissues or isolated macrophages were lysed with RIPA buffer (Beyotime Biotechnology). The protein concentration was assessed by a Bradford assay (Bio-Rad Laboratories). Protein samples (20 μg) were resolved using an 8% polyacrylamide gel and electrophoretically transferred to nitrocellulose membranes; the membranes were then blocked with nonfat milk in 0.1% Tween-20 in PBS for 1 h. Then, the membranes were incubated with primary antibodies (Table S2) at room temperature for 1.5 h, and after washing, the membranes were incubated with HRP-conjugated secondary antibodies. The specific signals were detected using an enhanced chemiluminescence Western blot detection system (Bio-Rad) after washing, following the manufacturer’s instructions. β-actin was used as the loading control.

### In vitro study of bone marrow-derived macrophages

NSE-BMP4 mice were sacrificed, and the hindlimbs were harvested to isolate the long bones (including the femur and tibia) in a biosafety hood. PBS injected with a syringe and needle was used to flush out the bone marrow from the femur and tibia. Red blood cells in the flushed bone marrow were lysed with RBC lysis buffer (NH_4_Cl (8.99 g·L^−1^) + KHCO_3_ (1 g·L^−1^) + Na_4_-EDTA (0.037 g·L^−1^)). F4/80^+^ macrophages were sorted using a MACS kit containing anti-mouse F4/80 microbeads (130-110-443, Miltenyi) according to the manufacturer’s instructions before the cells were cultured in RPMI-1640 with 10% serum replacement (10828028, Gibco) and 1% penicillin/streptomycin (MRC). Recombinant mouse M-CSF (50 ng·mL^−1^, Life Technologies)/GM-CSF (50 ng·mL^−1^, Thermo) was used for macrophage polarization. A recombinant mouse FetA protein (10 ng·mL^−1^, R&D) was added to GM-CSF to test whether FetA can change the polarization of macrophages. F4/80^+^ macrophages were further analyzed by flow cytometry using an anti-CD206 antibody diluted with flow cytometry buffer (0.5% BSA + 0.09% sodium azide in PBS) (see Fig. S21). Flow cytometry data were analyzed with FlowJo software (TreeStar, Inc.) or CytExpert (4.0 version, Beckman).

### Flow cytometry

Quantification of macrophages and myeloid cells was performed by flow cytometry. For macrophages, injured site cells of mouse muscle were harvested, washed and incubated for 20 min at 4 °C in phosphatase buffered saline (PBS) containing the following antibodies: F4/80-PECy5 (123112, Biolegend), CD16/32-FITC (101306, Biolegend), CD206-APC (141708, Biolegend), CD11b-FITC (101206, Biolegend), and Gr1-PE (108408, Biolegend). M1 macrophages were defined as CD16/32^+^/CD206^-^ and M2 macrophages were defined as CD206^+^. CD11b^+^/Gr1^+^ cells were considered myeloid cells.

### Statistical analysis

Unless otherwise noted, data are presented as the mean±s.d. of biological replicates (independent animals/independent experiments; the numbers (*n*) are specified in each figure legend). Unpaired two-tailed Student’s t tests were used to determine the significance of differences between two groups. *P* < 0.05 was considered statistically significant (^*^P < 0.05; ^**^P < 0.01, ^***^P < 0.001, and ^****^P < 0.000 1).

## Supplementary information

Supplementary figures

Supplementary movie 1

Supplementary movie 2

Supplementary movie 3

## Data Availability

The RNA-sequencing data are deposited in GEO (accession number: GSE161388). The clinical characteristics of all patients included in the present study are shown in Tables S1 and S2. All gene sets are shown in Table S3. The antibodies used are shown in Table S4. Full scans of all of the blots and gels are included in Figure S20. The data that support the findings of this study are available from the corresponding author upon request.
