# Correction To: Fetuin-A is an immunomodulator and a potential therapeutic option in BMP4-dependent heterotopic ossification and associated bone mass loss

**DOI:** 10.1038/s41413-022-00238-5

**Published:** 2022-11-15

**Authors:** Chen Kan, Jiazhao Yang, Haitao Fan, Yuanjuan Dai, Xingxing Wang, Rui Chen, Jia Liu, Xiangyue Meng, Wei Wang, Guiling Li, Jiao Zhou, Ya Zhang, Wanbo Zhu, Shiyuan Fang, Haiming Wei, Hong Zheng, Siying Wang, Fang Ni

**Affiliations:** 1 Department of Pathophysiology, School of Basic Medical Sciences, Anhui Medical University, Hefei, China; 2 Department of Hematology, The First Affiliated Hospital of USTC, The CAS Key Laboratory of Innate Immunity and Chronic Disease, School of Basic Medical Sciences, Division of Life Sciences and Medicine, University of Science and Technology of China, Hefei, China; 3 Institute of Immunology, University of Science and Technology of China, Hefei, China; 4 Department of Orthopaedics, The First Affiliated Hospital of USTC, Hefei, China; 5 Department of Orthopaedics, Fuyang Hospital of Anhui Medical University, Fuyang, China

**Keywords:** Bone, Metabolic diseases

Correction to: *Bone Research* 10.1038/s41413-022-00232-x, published online 27 October 2022

Following publication of this article^1^, it is noticed that a sentence “FetA was characterized as a decoy receptor of transforming growth factor beta (TGF-β)/BMP, suggesting that FetA could be useful to inhibit and/or prevent TGF-β/BMP-induced diseases, including heterotopic ossification^61,62,63,64^.” needs to be inserted in the section DISCUSSION. References 61–64 are also added.

In addition, the reference number “2019YFA0801801” should be changed to “2019YFA0801800” in the section ACKNOWLEDGEMENTS.

The original article^1^ was updated.
