# Supplementary material for: Fetuin-A is an immunomodulator and a potential therapeutic option in BMP4-dependent heterotopic ossification and associated bone mass loss
Source: Bone Res. 2022 Oct 27;10:62. doi: 10.1038/s41413-022-00232-x (PMC9605967; doi:10.1038/s41413-022-00232-x)

Supplementary information

Supplementary Tables 1-4

Supplementary Table 1, Clinical data of non-HO patients with injury

Patients' ID	Age	Gender	Types of trauma	Diagnosis
2481317	74	Female	Traffic accident	Left acetabular fracture
2464030	34	Female	Traffic accident	Left acetabular fracture
2644701	44	Female	Traffic accident	Right acetabular fracture
2883240	72	Female	Traffic accident	Left acetabular fracture
2880987	48	male	Traffic accident	Right acetabular fracture
2469526	36	male	Crush injury	Left acetabular fracture
2469820	37	male	Traffic accident	Right acetabular fracture
2642055	54	male	Falling accident	Right acetabular fracture
2706121	68	male	Falling accident	Right acetabular fracture
2630683	38	male	Traffic accident	Right acetabular fracture
2610610	44	male	Traffic accident	Left acetabular fracture

Supplementary Table 2, Clinical data of aHO patients

Patients' ID	Age	Gender	Types of trauma	Diagnosis	HO location
2459198	39	Male	Bicycle accident	Left acetabular fracture	Left elbow
2211874	56	Male	Walking slip	Left acetabular fracture	Right hip
2400075	28	Male	Traffic accident	Right acetabular fracture	Right hip and thigh
2835428	29	Male	Traffic accident	Left acetabular fracture	Right thigh
2412399	51	Male	Falling accident	Right acetabular fracture	Left hip
2476175	32	Male	Traffic accident	Left acetabular fracture	Left hip
2422687	52	Male	Traffic accident	Right acetabular fracture	Bilateral hip, right elbow
2882380	37	Female	Traffic accident	Right acetabular fracture	Left knee
2465733	52	Male	Traffic accident	Right acetabular fracture	Right thigh
0377126	61	Female	Walking slip	Right acetabular fracture	Right knee

Supplementary Table 3, Summary of qPCRprimers used in this study

Genes ID	Primers Sequence (5'-3')
Ifng	Forward: ATGAACGCTACACACTGCATC<br>Reverse: CCATCCTTTTGCCAGTTCCTC
Il6	Forward: TAGTCCTTCCTACCCCAATTTCC<br>Reverse: TTGGTCCTTAGCCACTCCTTC
Tnfa	Forward: CCCTCACACTCAGATCATCTTCT<br>Reverse: GCTCACACTCAGATCATCTTCT
Il4	Forward: GGTCTCAACCCCCAGCTAGT<br>Reverse: GCCGATGATCTCTCTCAAGTGAT
Il10	Forward: GCTCTTACTGACTGGCATGAG<br>Reverse: CGCAGCTCTAGGAGCATGTG
Il13	Forward: CCTGGCTCTTGCTTGCCTT<br>Reverse: GGTCTTGTGTGATGTTGCTCA

Supplementary Table 4, Summary of primary antibodies used in this study

Antibody	Dilution	Source	Identifier
Anti-mouse TNFSF11	1:200	Abcam	Ab45039
Anti-mouse F4/80	1:200	Thermo	14480815
Anti-mouse IL-6	1:200	Abcam	ab179570
Anti-human Fetuin-A	1:200	Thermo	PA5-51593
Anti-mouse Fetuin-A	1:200	Thermo	MA5-29650
Anti-mouse CD27	1:200	Abcam	ab175403
Anti-mouse CD40	1:200	Abcam	ab13545
Anti-mouse CTSK	1:200	Abcam	ab19027
Anti-mouse CD206	1:200	R&D	AF2535
Anti-mouse PD1	1:200	Thermo	MA5-27899
Anti-mouse TIM3	1:200	Abcam	ab185703
Anti-mouse IBA1	1:200	Thermo	PA5-18488
Anti-mouse CD16/32	1:200	CST	80366
Anti-mouse Activin A	1:200	Abcam	ab227234
Anti-mouse CXCL9	1:200	Abcam	Ab202961
Anti-mouse SDF1	1:200	Proteintech	17402-1-AP
Anti-mouse CXCR3	1:200	Proteintech	26756-1-AP
Anti-mouse COX2	1:200	Proteintech	66351-1-Ig
Anti-mouse mTOR	1:200	Thermo	PA5-20123
Anti-mouse CD3	1:200	eBioscience	11-0031-82
Anti-mouse CD4	1:200	eBioscience	42-0042-82
Anti-mouse CD8	1:200	eBioscience	14-0081-82
Anti-mouse Actin	1:200	Thermo	MA5-11866

Supplementary Figures 1-20

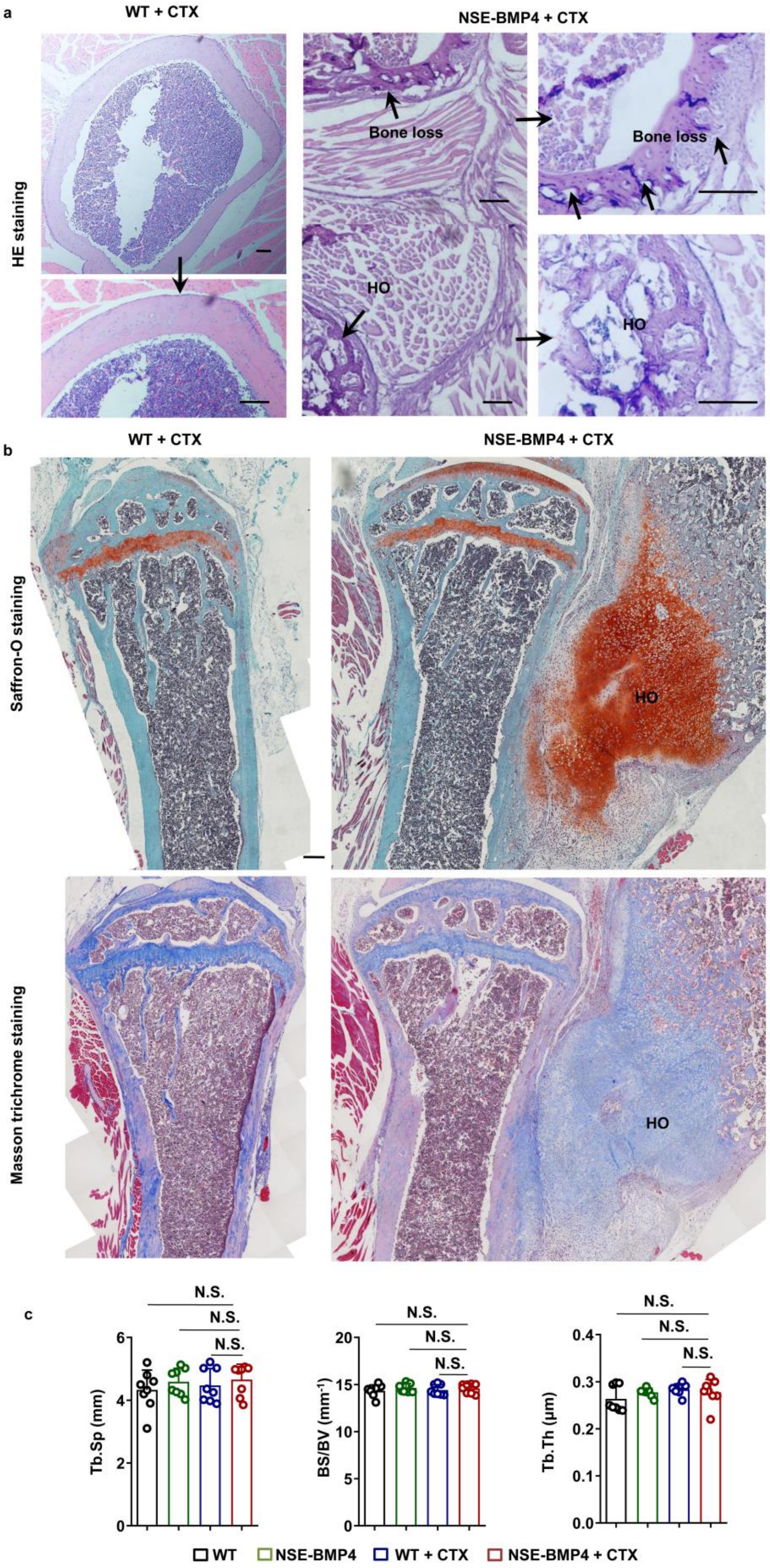

Supplementary Fig. 1 Histology and structural analysis of injured limbs in HO model mice. a, HE staining images of the histology of the tibia of WT and NSE-BMP4 mice following CTX injury. Scale bar, 200 μm. **b**, Saffron-O staining and Masson trichrome staining images of the tibia of WT and NSE-BMP4 mice following CTX injury. Scale bar, 200 μm. **c,** Statistical analysis of bone parameters, including the ratio of bone surface to bone volume (BS/BV), trabecular separation (Tb. Sp) and trabecular thickness (Tb. Th) in WT and NSE-BMP4 mice with or without injury (n=3). Data are presented as the mean ± s.d. of biological replicates. N.S. indicates no significance (unpaired two-tailed *t* test).

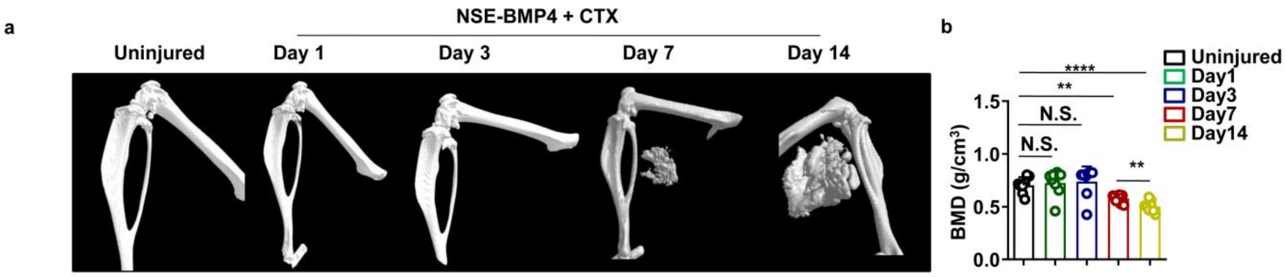

Supplementary Fig. 2 Structural analysis of injured tibia in HO model mice at different times post injury. a, b, Representative microCT images (**a**) and BMD analysis (**b**) of tibia from NSE-BMP4 mice with or without injury. n=8 per group. Data are presented as the mean ± s.d. of biological replicates. *P < 0.05, **P < 0.01, ****P < 0.0001, N.S. indicates no significance (unpaired two-tailed t test).

Supplementary Fig. 3 HE staining of injured tibia in HO model mice at 7 and 14 dpi.

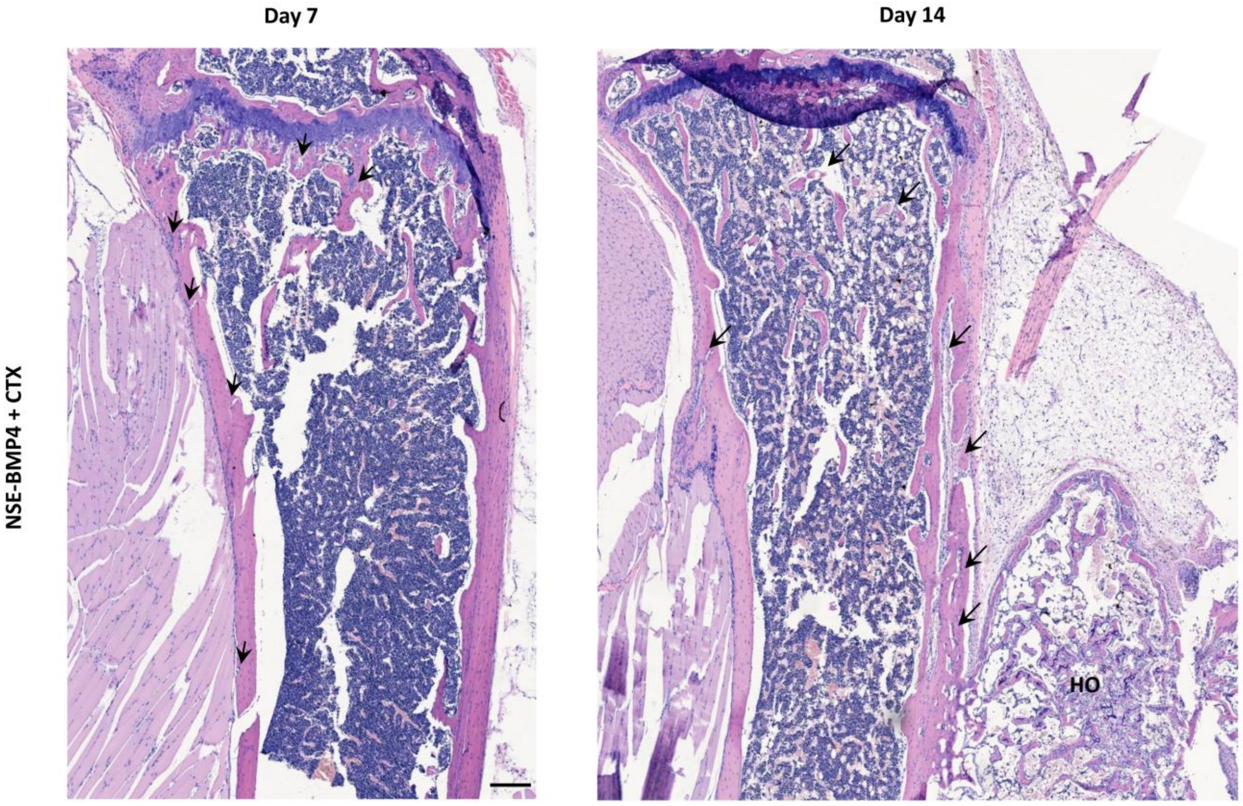

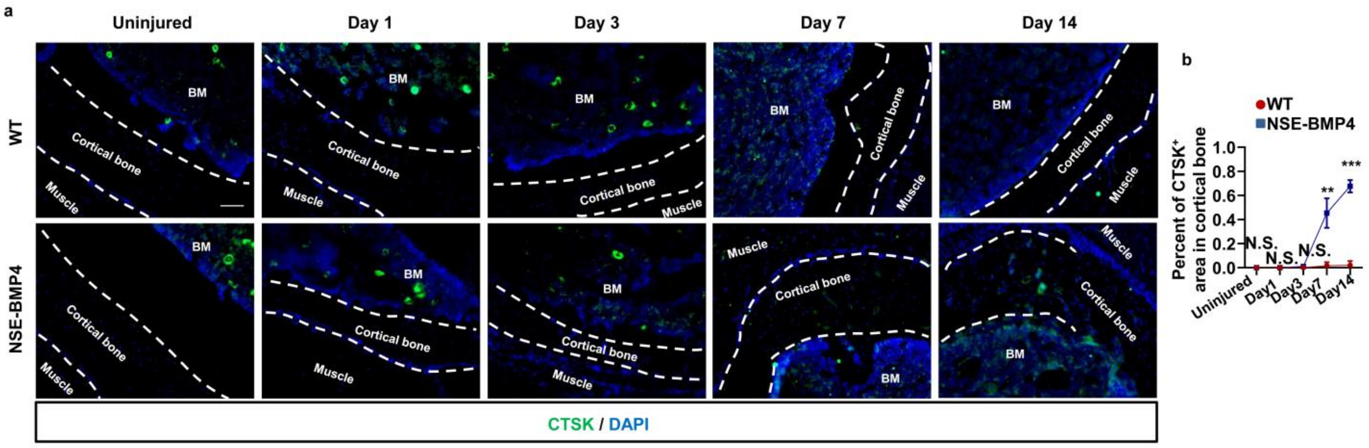

Supplementary Fig. 4 Immunofluorescence analysis of tibial bone close to HO at different times post injury. a, b, Representative immunostaining images and statistical analysis of CTSK in HO-adjacent cortical bone at 1, 3, 7 and 14 dpi. Data are presented as the mean ± s.d. of biological replicates. **P < 0.01, ***P < 0.001, N.S. indicates no significance (unpaired two-tailed t test).

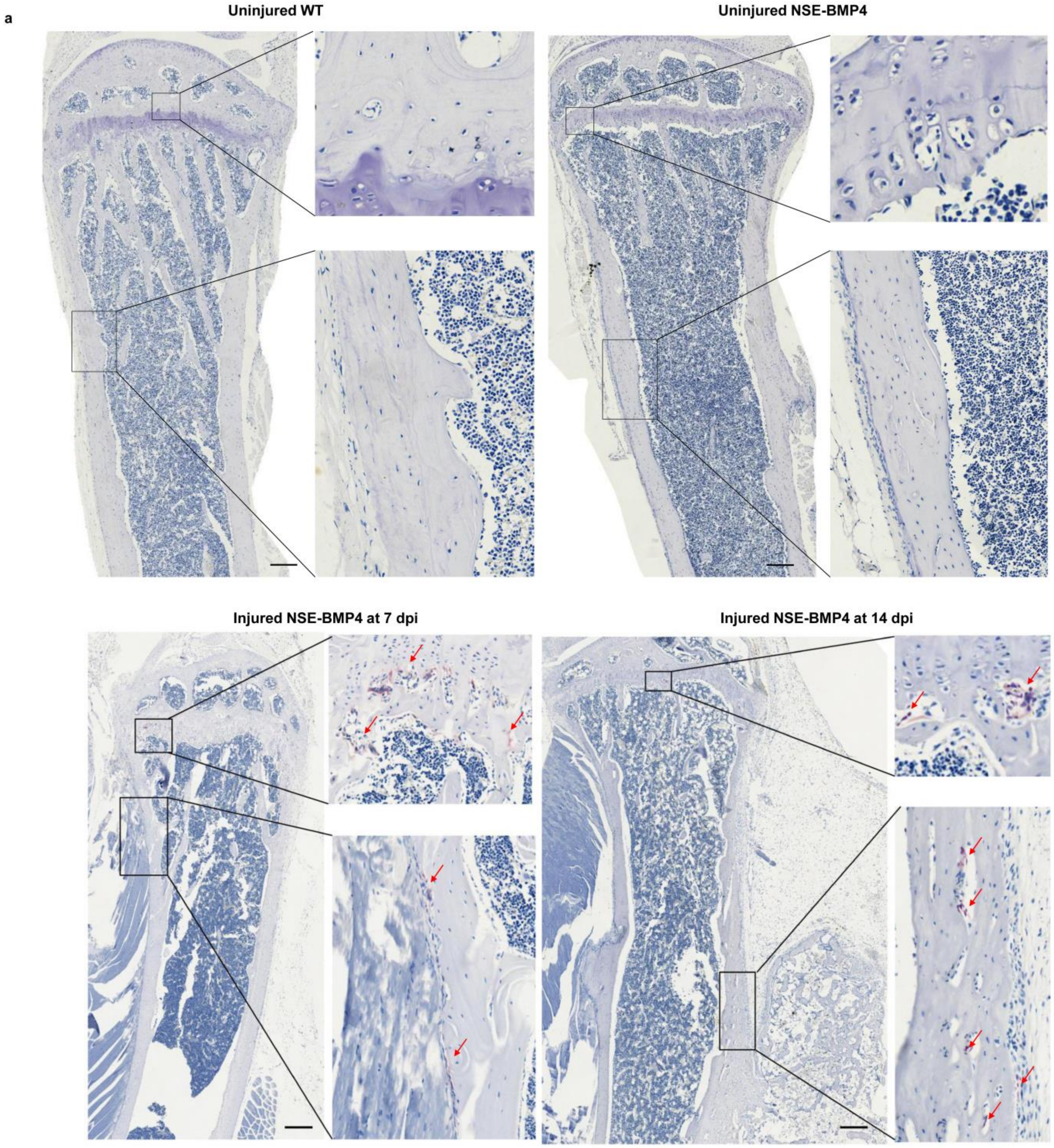

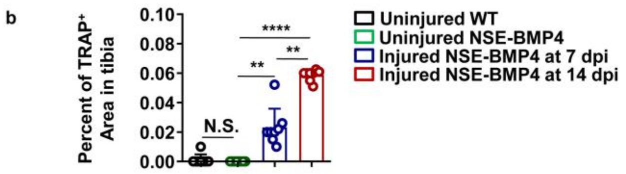

Supplementary Fig. 5 Cytochemical staining of tibial bone close to HO at different times post injury. a, b, Representative TRAP staining images and statistical analysis of the TRAP$^+$ area in the tibia of uninjured WT and NSE-BMP4 mice as well as the HO-adjacent tibia at 7 and 14 dpi in injured NSE-BMP4 mice. Scale bar, 200 μm. Data are presented as the mean ± s.d. of biological replicates. **P < 0.01, ****P < 0.0001, N.S. indicates no significance (unpaired two-tailed t test).

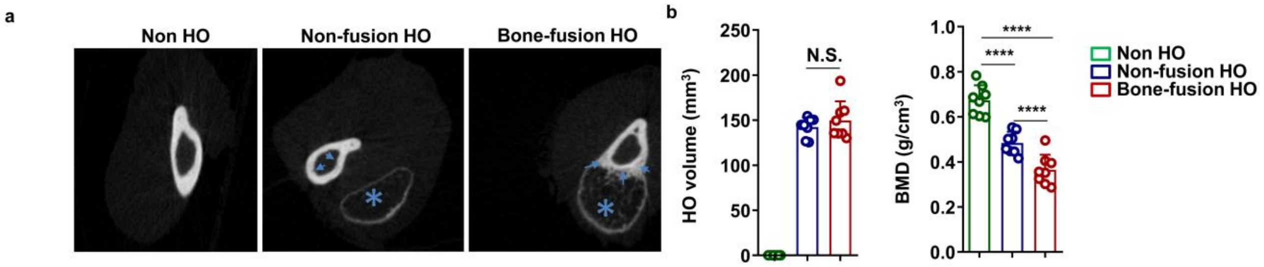

Supplementary Fig. 6 HO-bone fusion enhances bone mass loss. a, b, Representative microCT images of the tibia (**a**) and statistical analysis (**b**) of HO volume and BMD area in HO model mice at 14 dpi. Data are presented as the mean ± s.d. of biological replicates. ****P < 0.0001, N.S. indicates no significance (unpaired two-tailed t-test).

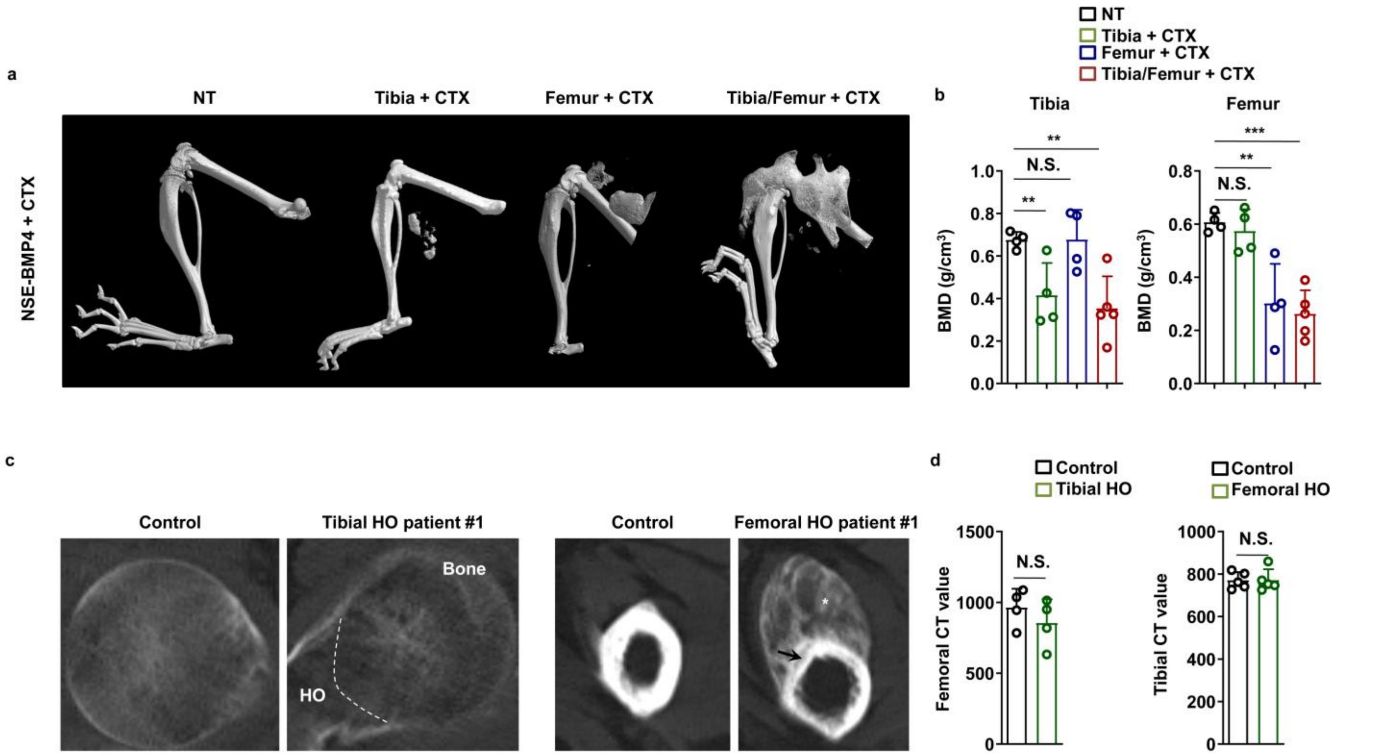

Supplementary Fig. 7 Tibial HO did not affect femur BMD, and femoral HO did not affect tibial BMD. a, Representative microCT image of the left hindlimb of NSE-BMP4 mice with or without injury adjacent to the tibia, femur or both sites. **b**, Statistical analysis of the tibial or femoral BMD in NSE-BMP4 mice with or without injury (n=4-5 per group). Data are presented as the mean ± s.d. of biological replicates. $^{**}P < 0.01$, $^{***}P < 0.001$, N.S. indicates no significance (unpaired two-tailed *t* test). **c**, Representative images of bone from tibial or femoral HO patients. **d**, Statistical analysis of the CT values of the tibia or femur in tibial and femoral HO patients. Data are presented as the mean ± s.d. of biological replicates. N.S. indicates no significance (unpaired two-tailed *t* test).

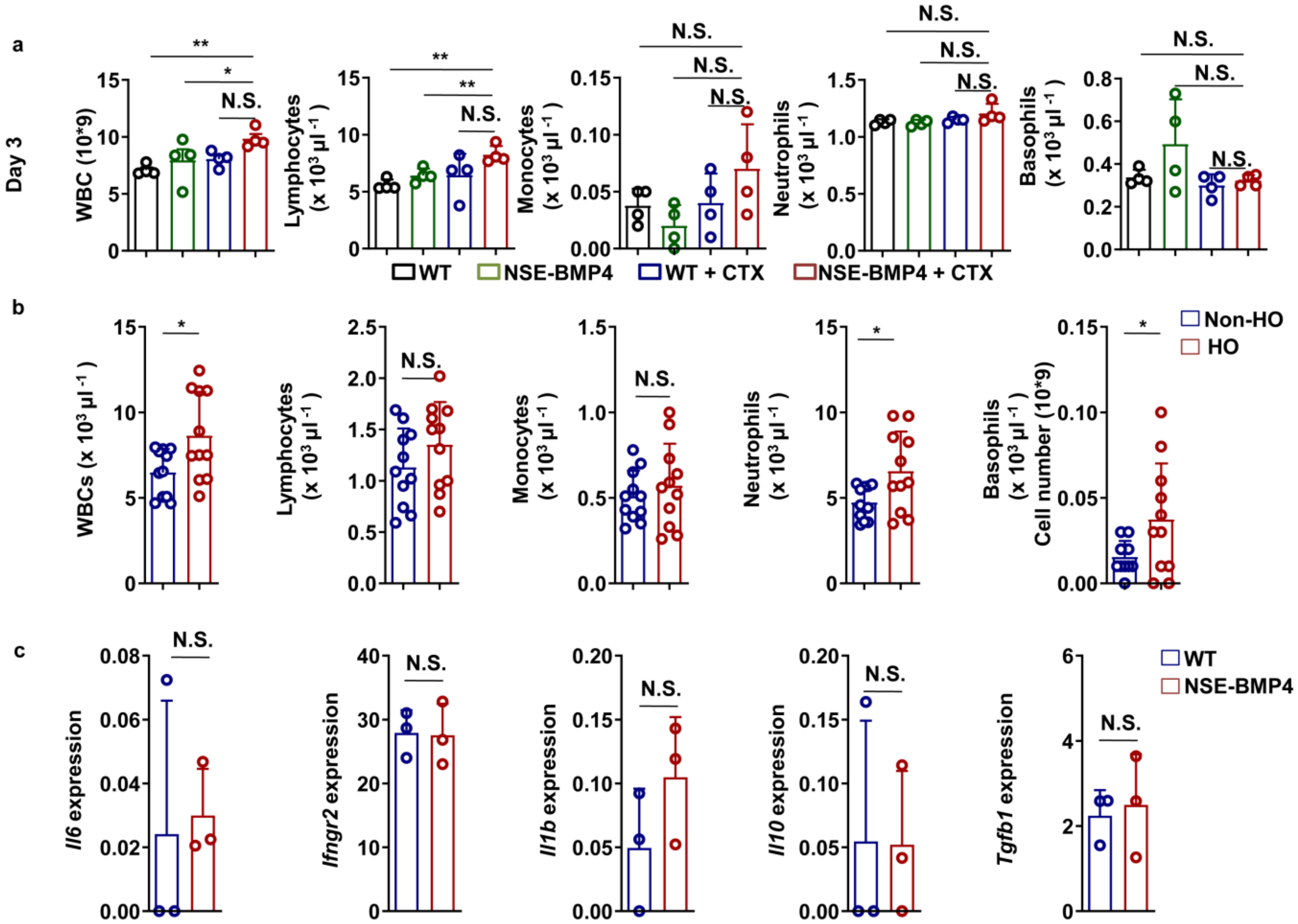

Supplementary Fig. 8 Analysis of blood cells of HO model mice and patients and inflammatory cytokine expression in NSE-BMP4 mouse muscle. a, Statistical analysis of peripheral blood cells in WT and NSE-BMP4 mice at 3 dpi**.** Data are presented as the mean ± s.d. of biological replicates. *P< 0.05, **P< 0.01, N.S. indicates no significance (unpaired two-tailed *t* test). **b**, Statistical analysis of the absolute numbers of WBCs, lymphocytes, monocytes, neutrophils and basophils in HO and non-HO patients. Data are presented as the mean ± s.d. of biological replicates. *P< 0.05, **P< 0.01, N.S. indicates no significance (unpaired two-tailed *t* test). **c,** Statistical analysis of the mRNA expression of inflammatory cytokines in uninjured muscle from WT and NSE-BMP4 mice. Data are presented as the mean ± s.d. of biological replicates. N.S. indicates no significance (unpaired two-tailed *t* test).

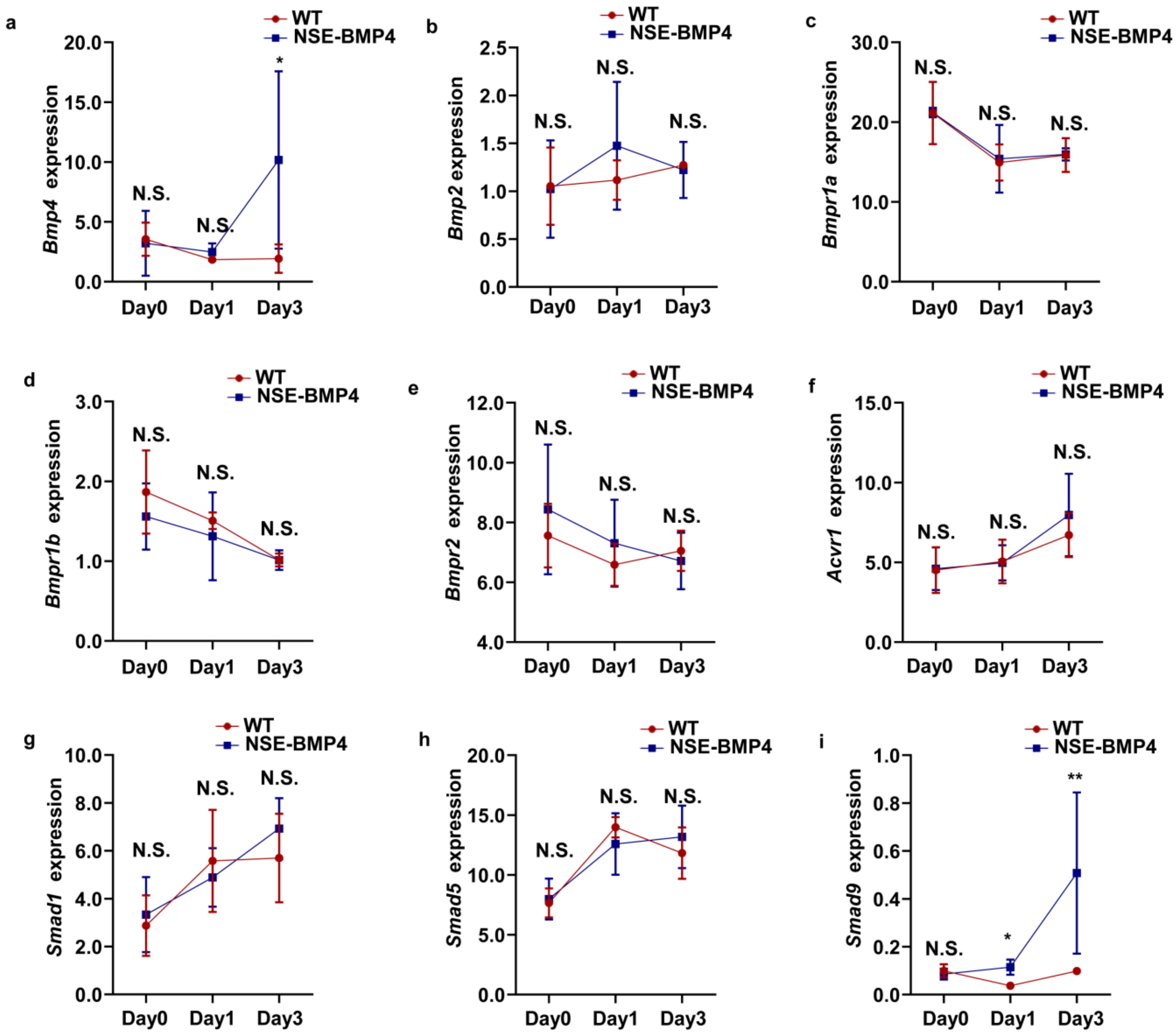

Supplementary Fig. 9 Analysis of BMP signaling-associated gene expression in injured muscle of WT and NSE-BMP4 mice. a, b, Statistical analysis of *Bmp4* and *Bmp2* expression in injured muscle of WT or NSE-BMP4 mice. Data are presented as the mean ± s.d. of biological replicates. *P< 0.05, N.S. indicates no significance (unpaired two-tailed *t* test). **c-f**, Statistical analysis of the expression of *Bmpr1a* (**c**), *Bmpr1b* (**d**), *Bmpr2* (**e**) and *Acvr1* (**f**) in injured muscle of WT or NSE-BMP4 mice. Data are presented as the mean ± s.d. of biological replicates. N.S. indicates no significance (unpaired two-tailed *t* test). **g- i**, Statistical analysis of the expression of *Smad1* (**g**), *Smad5* (**h**) and *Smad9* (**i**) in injured muscle of WT or NSE-BMP4 mice. Data are presented as the mean ± s.d. of biological replicates. *P< 0.05, **P< 0.01, N.S. indicates no significance (unpaired two-tailed *t* test).

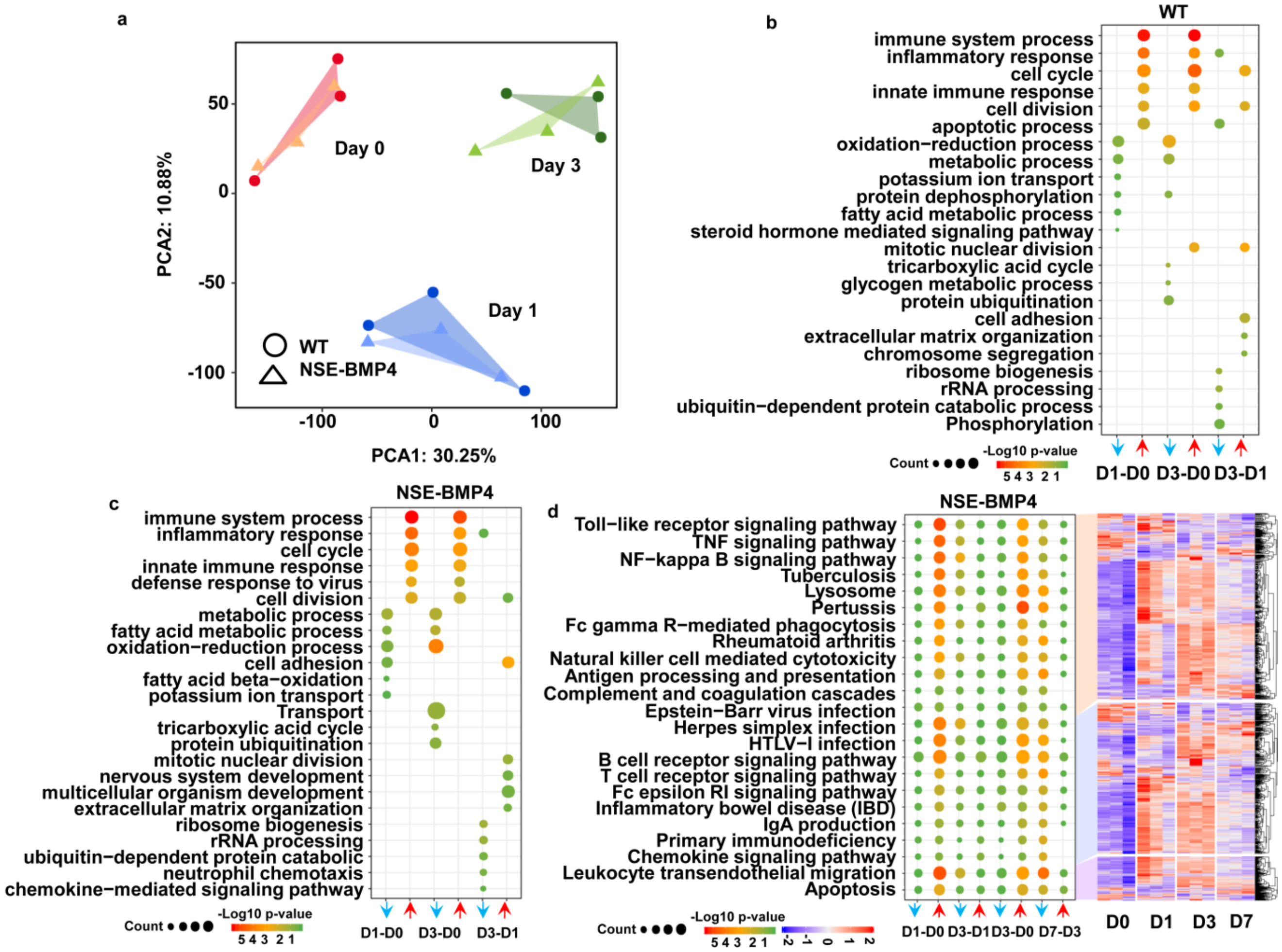

Supplementary Fig. 10 RNA-Seq analysis of immune-associated genes in injured muscle of WT and NSE-BMP4 mice. a, Principal component analysis of the levels of correlation and reproducibility of gene expression data among all samples. The same color represents biological duplicates of the same stage. **b, c,** GO enrichment analysis of DEGs in WT (**b**) and NSE-BMP4 (**c**) mice at different time points. Blue arrows indicate the upregulated genes, and downregulated genes are represented by red arrows. Dot size indicates the number of DEGs enriched in each term. **d**, KEGG pathway analysis and a heatmap of biological alterations in immunity in HO lesions at different time points after injury.

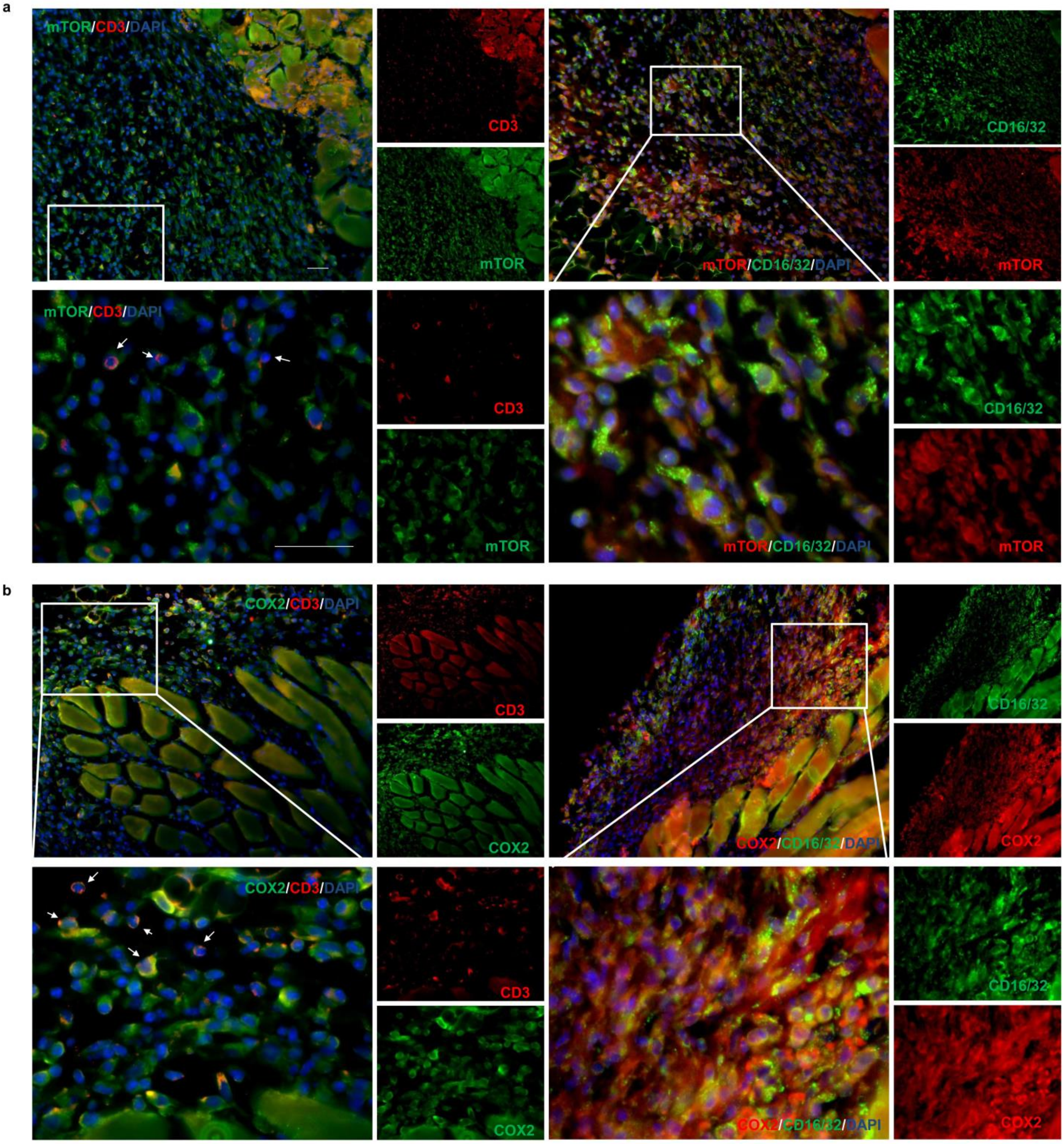

Supplementary Fig. 11 Both CD3$^+$ T cells and CD16/32$^+$ M1 macrophages expressed mTOR and COX2 in the injured tibial muscle of NSE-BMP4 mice at 1 dpi. **a**, Representative immunofluorescence staining images of mTOR, CD3 and CD16/32 in the injured sites of NSE-BMP4 mice at 1 dpi. Scale bar, 200 μm. **b**, Representative immunofluorescence staining images of COX2, CD3 and CD16/32 in the injured sites of NSE-BMP4 mice at 1 dpi. Scale bar, 200 μm.

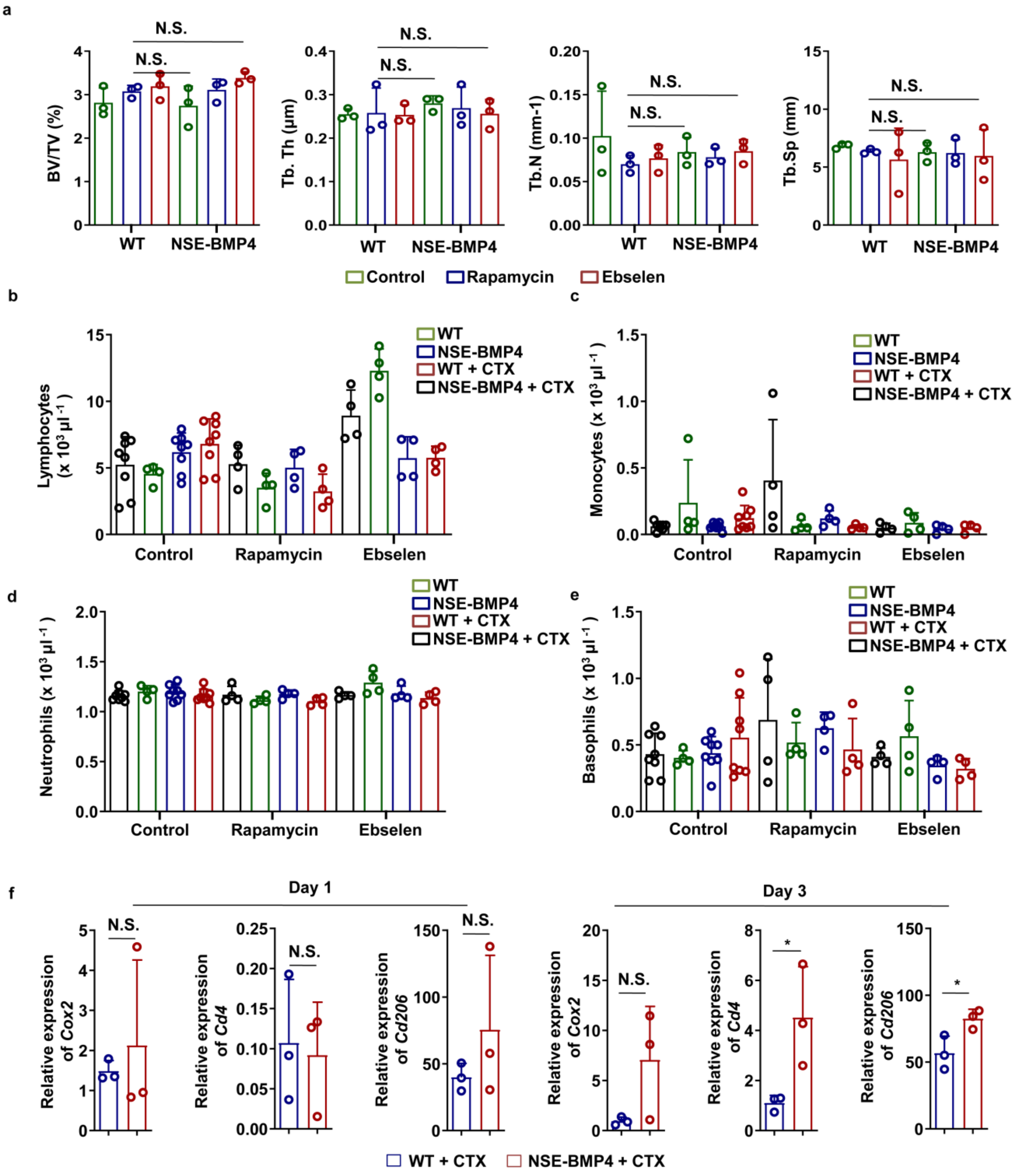

Supplementary Fig. 12 Alterations in bone parameters, blood cells and lesional immune-related genes in HO model mice with or without anti-inflammatory drug treatment. a, Statistical analysis of the bone structure in uninjured and injured NSE-BMP4 mice treated with or without anti-inflammatory drugs. Data are presented as the mean ± s.d. of biological replicates. N.S. indicates no significance (unpaired two-tailed *t* test). **b-e**, Statistical analysis of lymphocytes (**b**), monocytes (**c**), neutrophils (**d**) and basophils (**e**) in injured WT and NSE-BMP4 mice treated with or without anti-inflammatory drugs. Data are presented as the mean ± s.d. of biological replicates. ***P*< 0.01, N.S. indicates no significance (unpaired two-tailed *t* test). **f**, Statistical analysis of the expression of *Cox2, Cd4 and Cd206* in the injured muscle of WT and NSE-BMP4 mice evaluated via RNA-seq at 1 and 3 dpi. **P*< 0.05, N.S. indicates no significance (unpaired two-tailed *t* test).

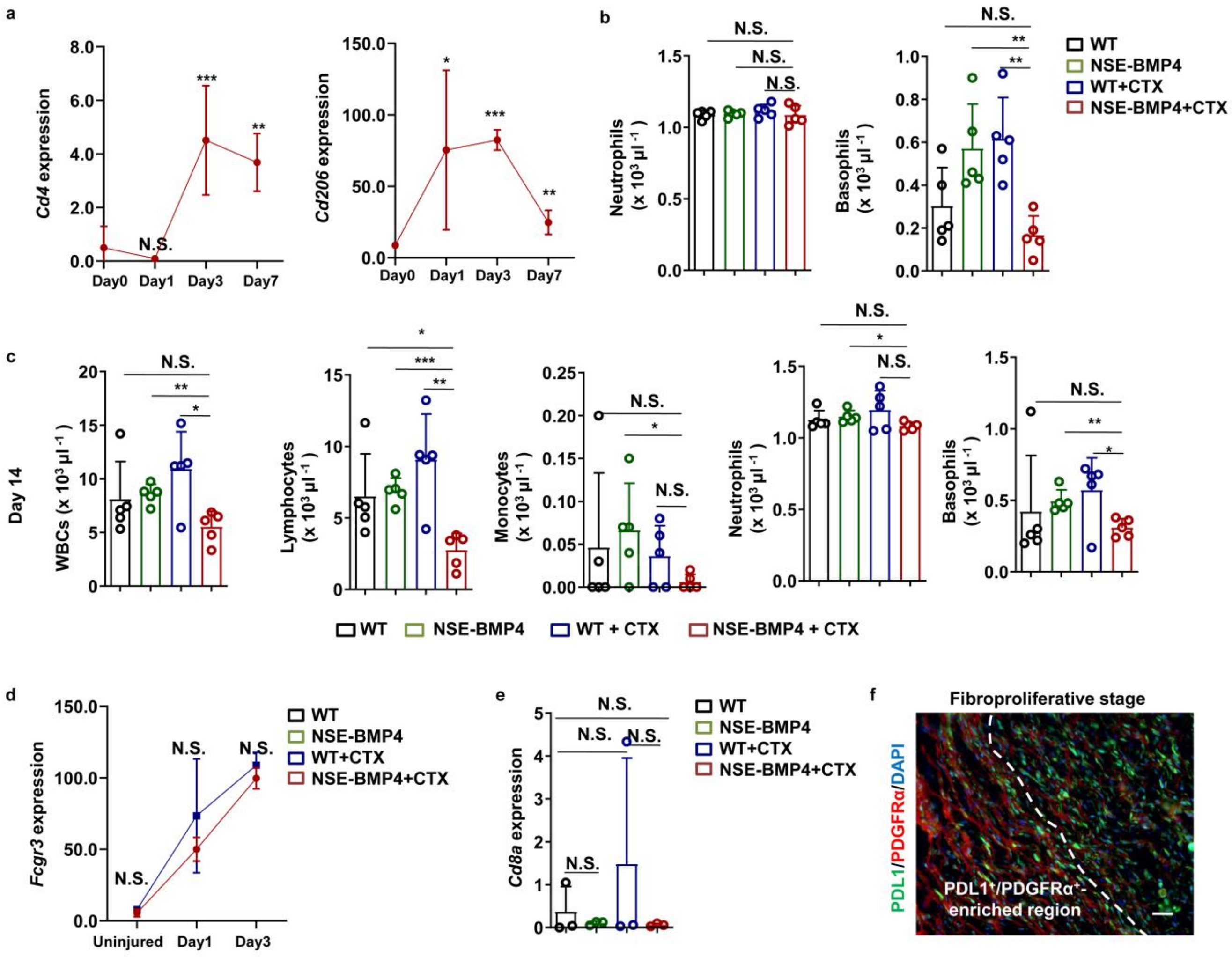

Supplementary Fig. 13 Analysis of immunity-associated genes, blood cells and PDL1 in NSE-BMP4 mice at different postinjury times. a, Statistical analysis of the expression of *Cd4 and Cd206* in injured muscle of NSE-BMP4 mice at different postinjury times. **b,** Statistical analysis of neutrophils and basophils in WT and NSE-BMP4 mice at 7 dpi (n=5 per group). Data are representative of two independent experiments. Data are presented as the mean ± s.d. of biological replicates. *P< 0.05, **P< 0.01, N.S. indicates no significance (unpaired two-tailed *t* test). **c,** Statistical analysis of total WBCs, lymphocytes, monocytes, neutrophils and basophils in WT and NSE-BMP4 mice at 14 dpi (n=5 per group). Data are representative of two independent experiments. Data are presented as the mean ± s.d. of biological replicates. * P< 0.05, ** P< 0.01, N.S. indicates no significance (unpaired two-tailed *t* test). **d**, **e,** Statistical analysis of the expression of *Fcgr3* and *Cd8a* in injured muscle of NSE-BMP4 mice. Data are presented as the mean ± s.d. of biological replicates. N.S. indicates no significance (unpaired two-tailed *t* test). **f**, Representative image of PDL1$^+$ and PDGFRα$^+$ cells in HO lesions at 3 dpi. Scale bar, 200 μm.

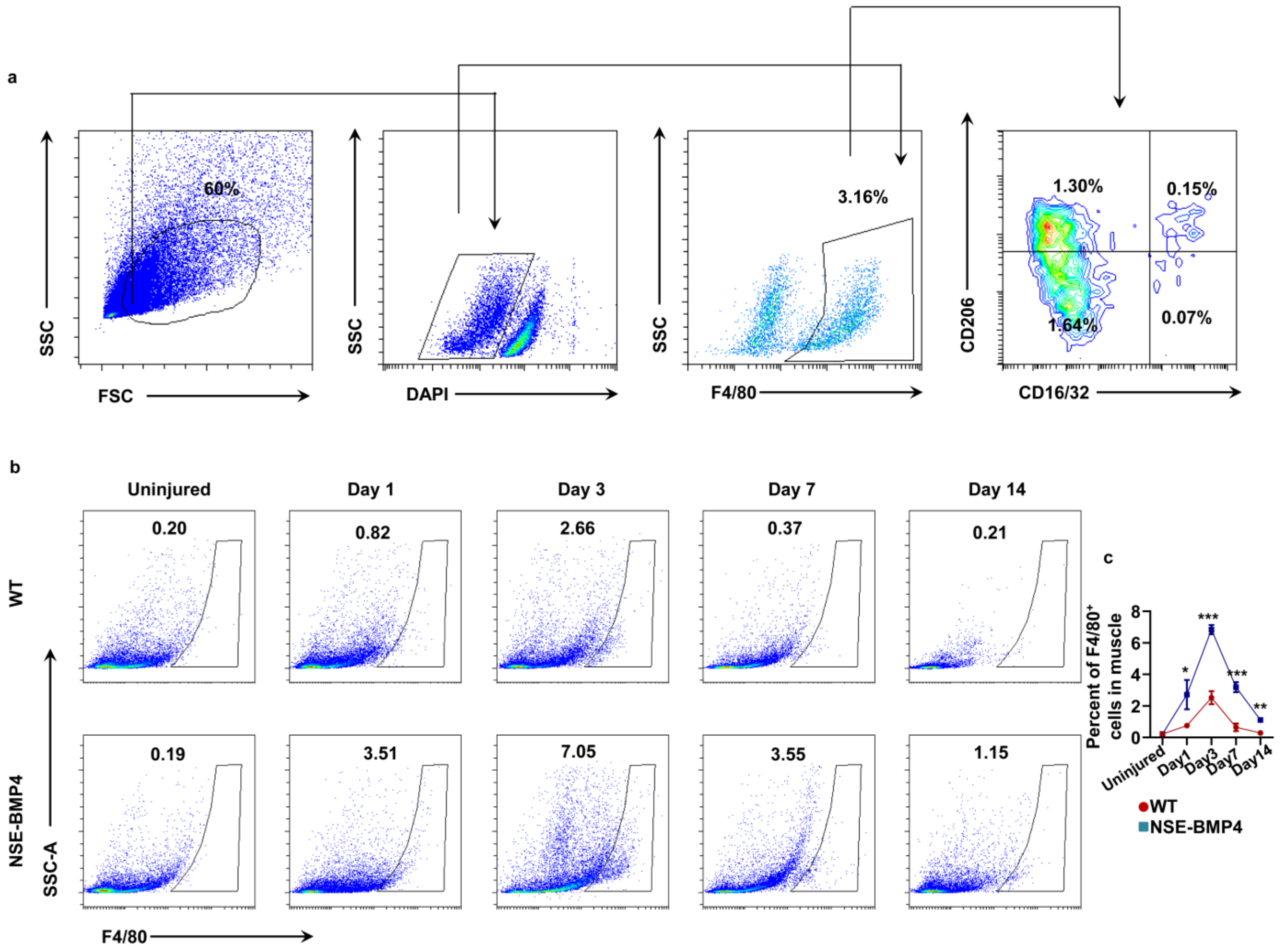

Supplementary Fig. 14 Representative flow cytometry images of macrophages. a, Representative gating strategies used in flow cytometry analyses for Figure 4d. **b, c,** Representative flow cytometry images and statistical analysis of F4/80$^+$ cells in uninjured and injured tibial muscle. Data are presented as the mean ± s.d. of biological replicates. *P< 0.05, **P< 0.01, ***P< 0.001 (unpaired two-tailed t test).

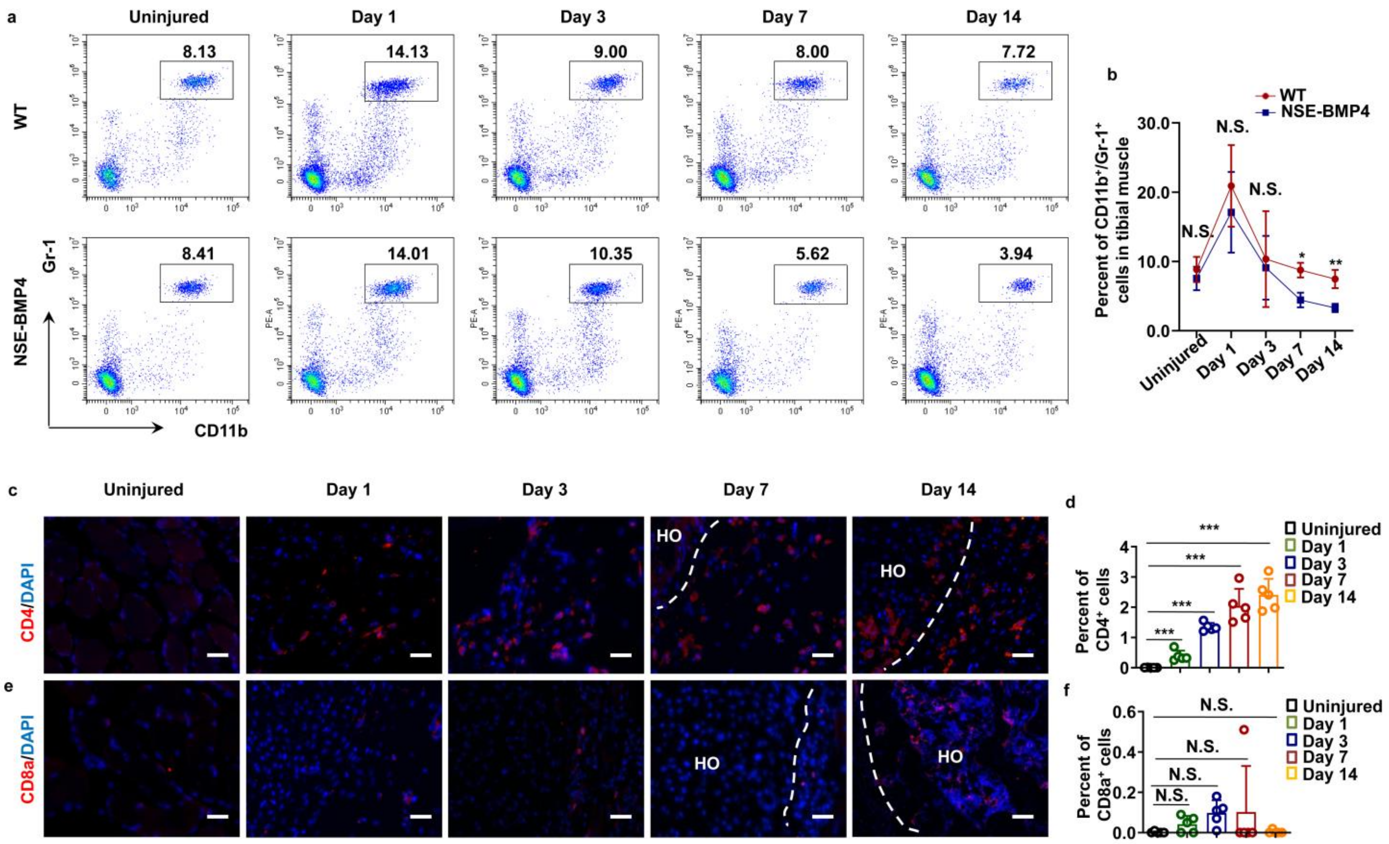

Supplementary Fig. 15 Analysis of myeloid cells and lymphocytes in injured muscle of NSE-BMP4 mice at different postinjury times. **a**, **b,** Representative FCM images (**a**) and statistical analysis (**b**) of myeloid cells in injured muscle of WT and NSE-BMP4 mice. **c, d,** Representative immunostaining images and statistical analysis of $CD4^+$ cells in injured muscle of NSE-BMP4 mice at different post injury times. Scale bar, 200 μm. **e, f,** Representative immunostaining images and statistical analysis of $CD8a^+$ cells in injured muscle of NSE-BMP4 mice at different post injury times. Scale bar, 200 μm.

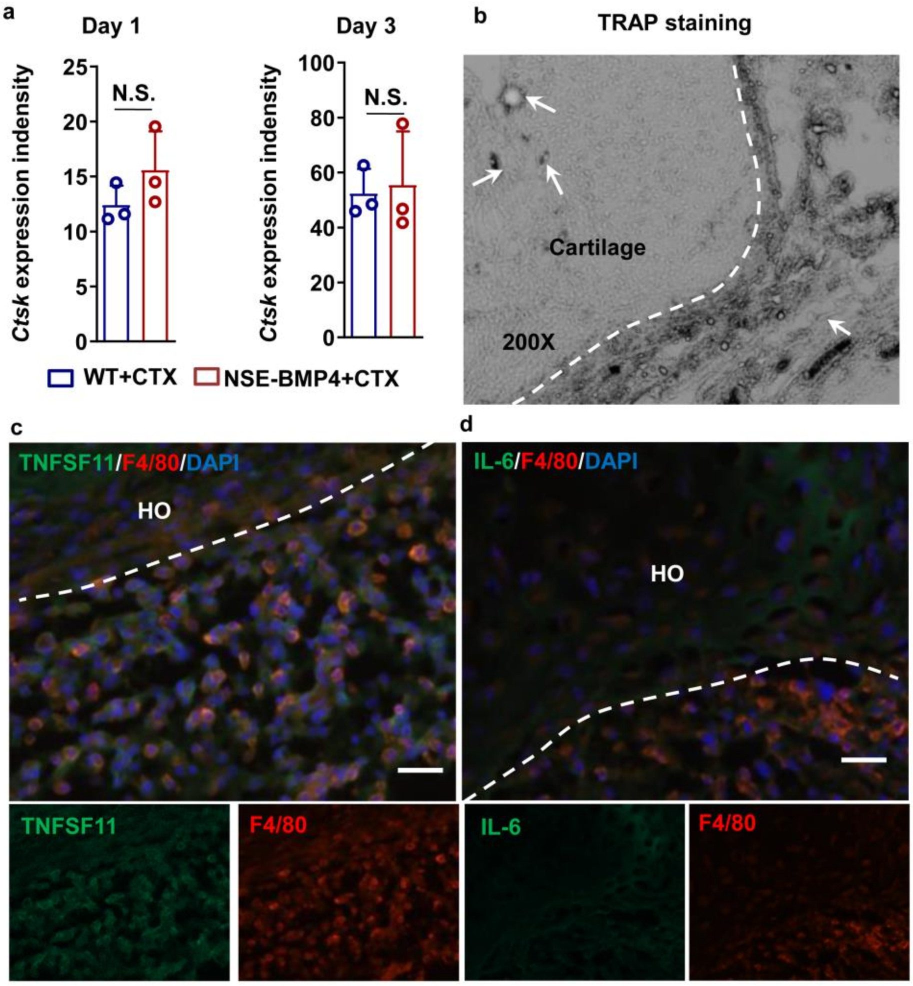

Supplementary Fig. 16 Osteoclasts were not generated in HO lesions at 3 dpi. a, Statistical analysis of *Ctsk* expression in the tibial muscle of WT and NSE-BMP4 mice at 1 or 3 dpi evaluated via RNA-seq. Data are presented as the mean ± s.d. of biological replicates. **P*< 0.05, ***P*< 0.01, N.S. indicates no significance. **b**, Representative image of TRAP$^+$ cells in HO lesions at 3 dpi. Images were captured with a 200 × lens. **c**, Representative image of TNFSF11$^+$ and F4/80$^+$ cells in HO lesions at 3 dpi. Scale bar, 200 μm. **d**, Representative image of IL-6$^+$ and F4/80$^+$ cells in HO lesions at 3 dpi. Scale bar, 200 μm.

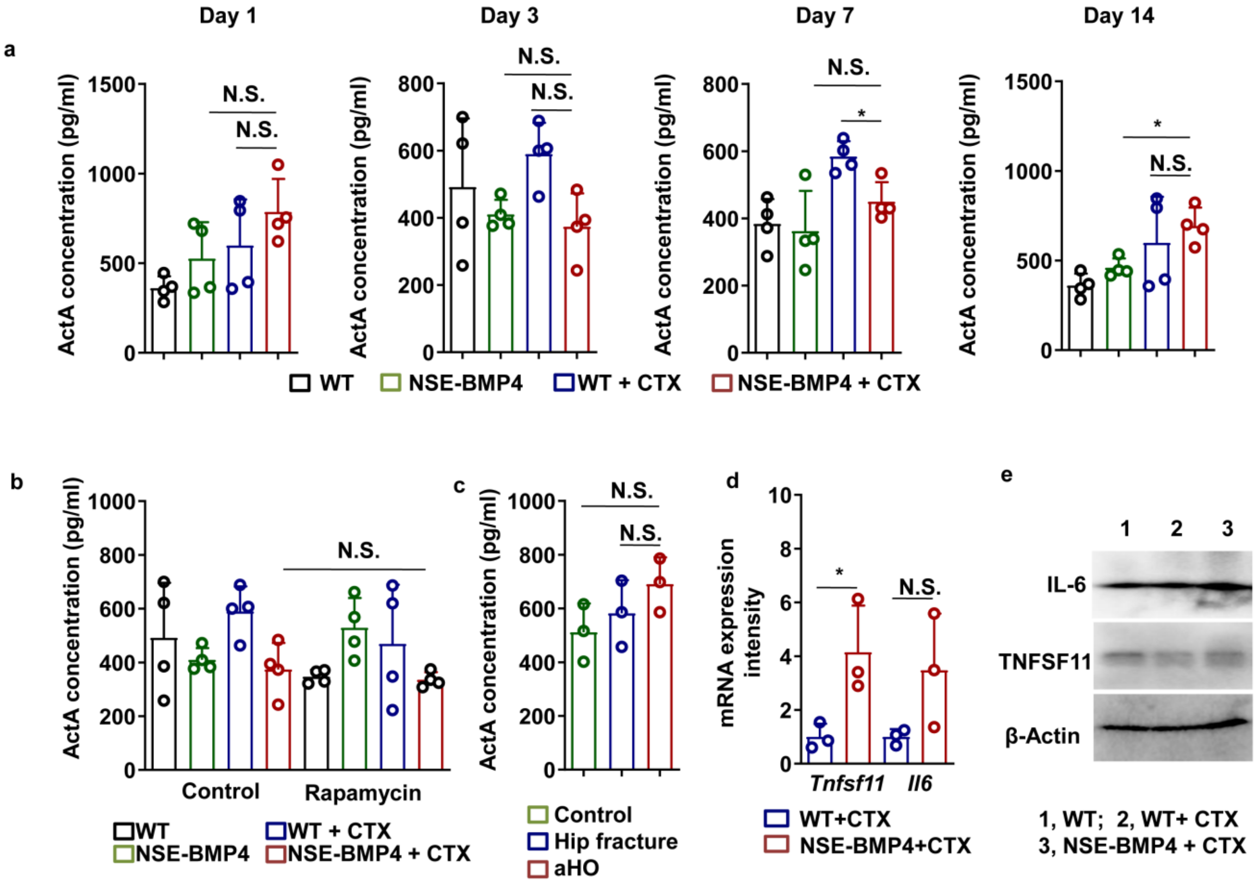

Supplementary Fig. 17 ActA and osteoclast-associated genes were unrelated to injury-induced HO with bone loss. a, Statistical analysis of ActA in the blood of WT and NSE-BMP4 mice at different time points after injury (n=4). Data are presented as the mean ± s.d. of biological replicates. **P*< 0.05, N.S. indicates no significance (unpaired two-tailed *t* test). **b**, Statistical analysis of ActA in the blood of WT and NSE-BMP4 mice following rapamycin treatment for 2 weeks (n=4). Data are presented as the mean ± s.d. of biological replicates. N.S. indicates no significance (unpaired two-tailed *t* test). **c**, Statistical analysis of ActA in the blood of aHO and non-HO patients (n=3). Data are presented as the mean ± s.d. of biological replicates. N.S. indicates no significance (unpaired two-tailed *t* test). **d**, Statistical analysis of the expression of *Tnfsf11* and *Il6* in the injured tibial muscle of WT and NSE-BMP4 mice. Data are presented as the mean ± s.d. of biological replicates. **P*< 0.05, N.S. indicates no significance (unpaired two-tailed *t* test). **e**, Western blot analysis of TNFSF11 and IL-6 expression in normal bone in proximity to the tibial muscle of WT and NSE-BMP4 mice with or without injury at 3 dpi.

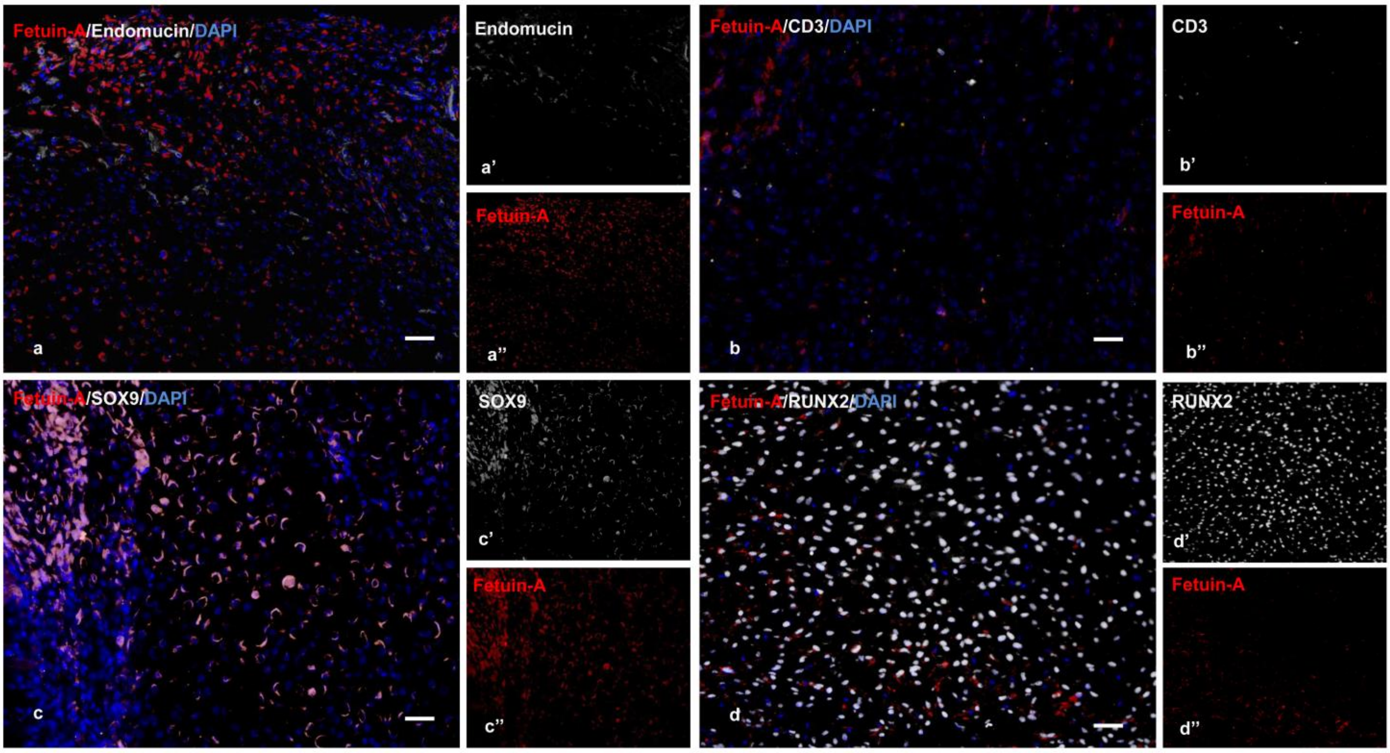

Supplementary Fig. 18 FetA was expressed in chondrocytes but not in vascular endothelial cells, T cells or osteoblasts at 7 dpi. a-a'', Representative images of costaining between FetA and endomucin in HO lesions at 7 dpi. Scale bar, 200 µm. **b-b''**, Representative images of costaining between FetA and CD3 in HO lesions at 7 dpi. Scale bar, 200 µm. **c-c''**, Representative images of costaining between FetA and SOX9 in HO lesions at 7 dpi. Scale bar, 200 µm. **d-d''**, Representative images of costaining between FetA and RUNX2 in HO lesions at 7 dpi. Scale bar, 200 µm.

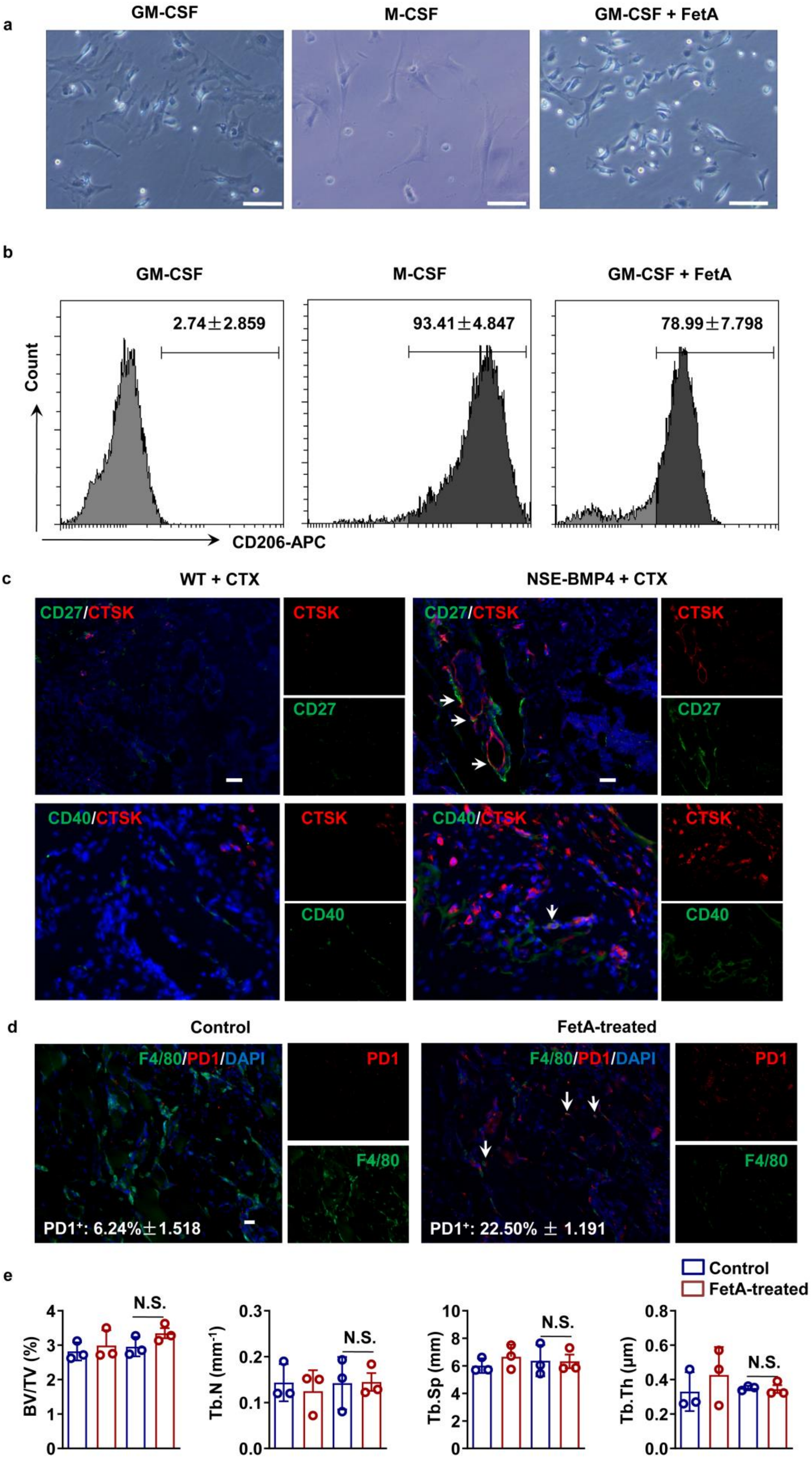
a
GM-CSF
M-CSF
GM-CSF + FetA
b
GM-CSF
M-CSF
GM-CSF + FetA
2.74±2.859
93.41±4.847
78.99±7.798
Count
CD206-APC
c
WT + CTX
NSE-BMP4 + CTX
CD27/CTSK
CTSK
CD27
CD40/CTSK
CTSK
CD40
d
Control
FetA-treated
F4/80/PD1/DAPI
PD1
F4/80
PD1+: 6.24%±1.518
PD1+: 22.50% ± 1.191
e
Control
FetA-treated
N.S.
BV/TV (%)
Tb.N (mm-1)
Tb.Sp (mm)
Tb.Th (μm)

Supplementary Fig. 19 FetA induced PD1 expression and macrophages to polarize into the M2 state, and loss of FetA promoted stimulatory IC molecule expression. a, The typical morphological features of different types of macrophages induced by GM-CSF, M-CSF or GM-CSF + FetA. **b** Representative FCM images and statistical analysis of the population of $CD206^+$ macrophages following treatment with GM-CSF, M-CSF and FetA. **c**, Immunostaining images of the distributions of $CD27^+$/$CTSK^+$ and $CD40^+$/$CTSK^+$ osteoclasts in the bone adjacent to HO lesions at 7 dpi. Scale bar, 200 μm. **d,** Representative costaining image of F4/80 and PD1 in injured muscle of NSE-BMP4 mice with or without FetA treatment. Scale bar, 200 μm. **e**, Statistical analysis of bone parameters of injured NSE-BMP4 mice with or without FetA treatment. Data are presented as the mean ± s.d. of biological replicates. N.S. indicates no significance (unpaired two-tailed *t* test).

Supplementary Fig. 20

Uncropped scans for Figure 7b

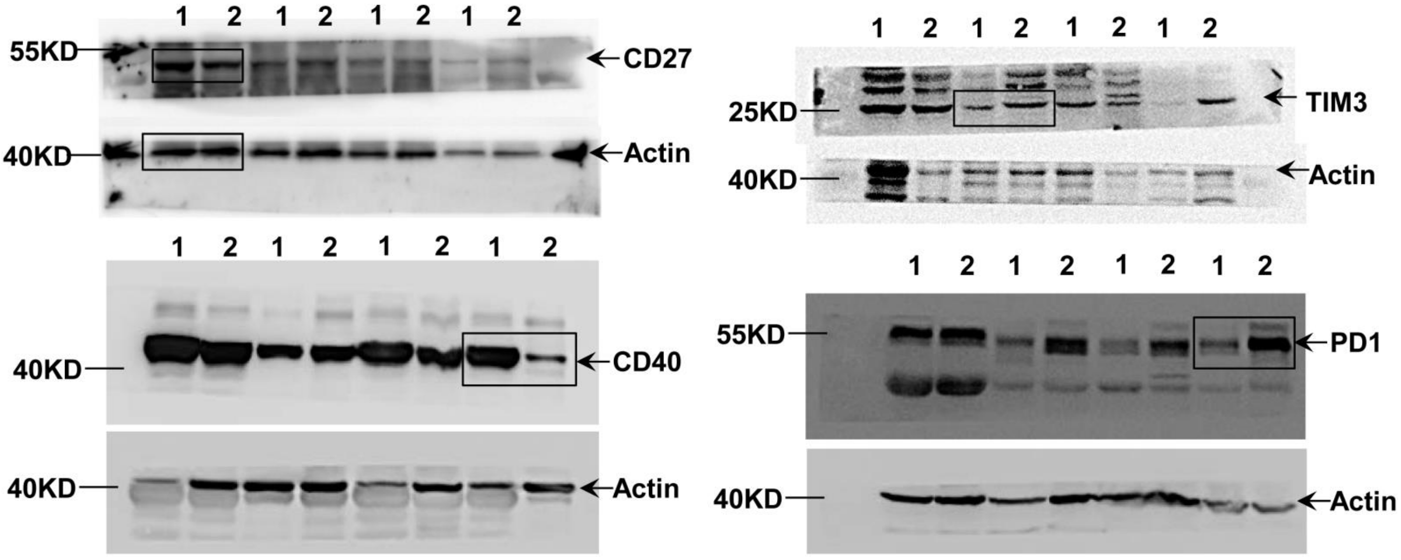

1: GM-CSF, 2:GM-CSF+FetA

Uncropped scans for Supplementary Figure 17e

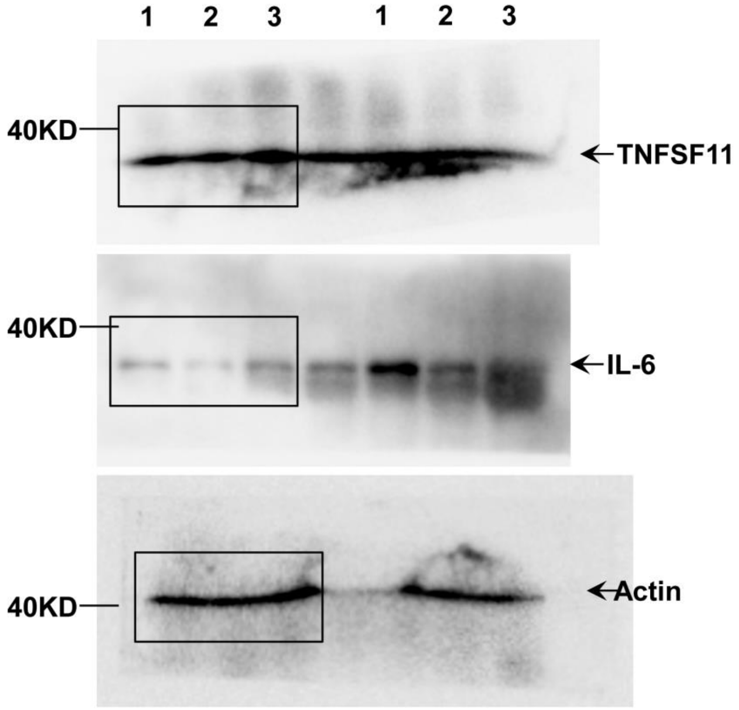

1, WT; 2, WT+ CTX
3, NSE-BMP4 + CTX

Supplementary Fig. 21

Representative gating strategies used in flow cytometry analyses for Figure 7a

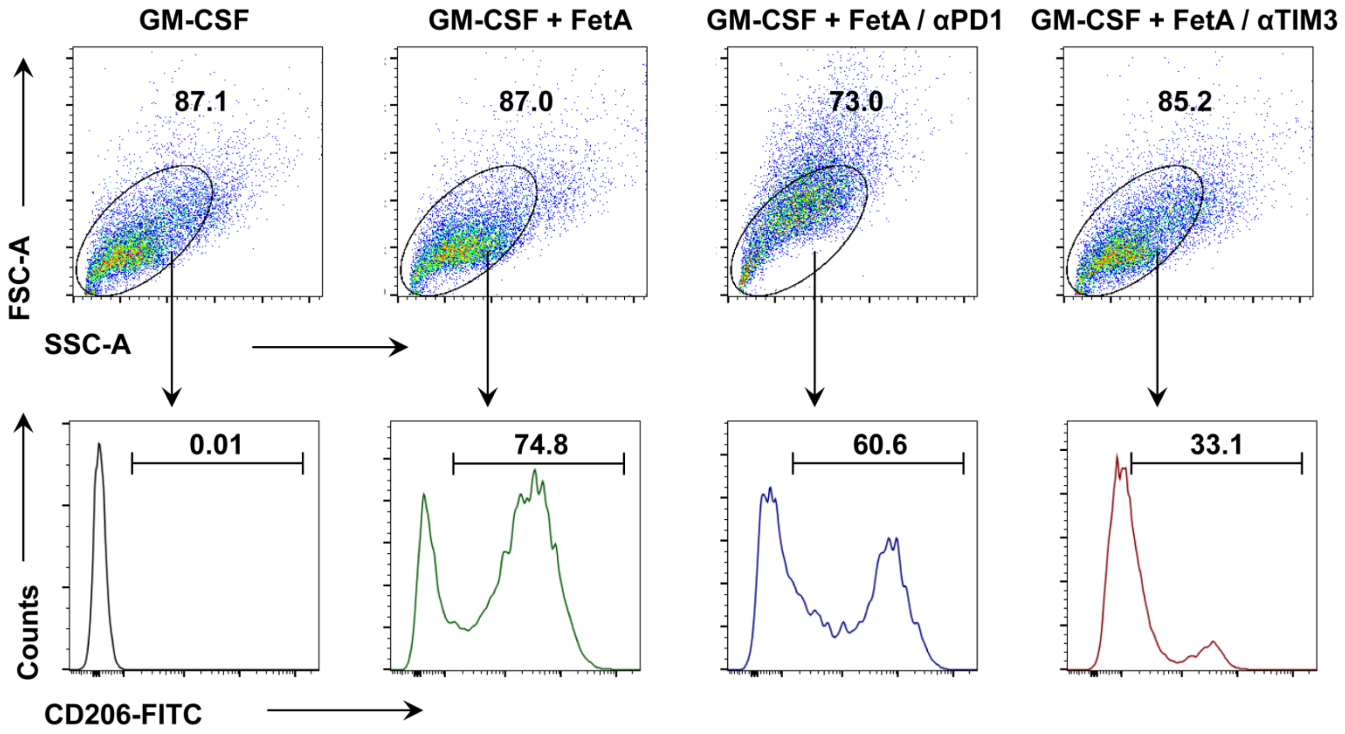

Supplement: Supplementary file 1 — Supplementary figures [file 41413_2022_232_MOESM1_ESM.pdf]
